# Dihydromyricetin Is Associated with Healthspan-Related Phenotypes and Neuroprotection in *Caenorhabditis elegans*, Alongside Redox and Mitochondrial Quality-Control Changes

**DOI:** 10.3390/antiox15101251

**Published:** 2026-09-30

**Authors:** Yong-Ha Jo, Hannah Cho, Dong-Sung Lee, Jeong Hoon Cho

**Affiliations:** 1 Research Institute of Pharmaceutical Sciences, College of Pharmacy, Chosun University, Gwangju 61452, Republic of Korea; 2 Honam Regional Future Drug Development Convergence Education & Research Division (BK21 FOUR), College of Pharmacy, Chosun University, Gwangju 61452, Republic of Korea; 3 Department of Biological Sciences, Chonnam National University, Gwangju 61186, Republic of Korea; 4 Department of Biology Education, Chosun University, Gwangju 61452, Republic of Korea

**Keywords:** dihydromyricetin, *Caenorhabditis elegans*, oxidative stress, mitochondrial quality control, mitochondrial trafficking, neuroprotection, healthspan-related phenotypes, lipid metabolism

## Abstract

Dihydromyricetin (DHM), a dietary flavanonol known for its antioxidant and neuroprotective properties, has been connected to mitochondrial homeostasis, yet how it engages mitochondrial quality control during aging has not been resolved. This study examined DHM’s effects on lifespan, neuronal integrity, resistance to oxidative stress, mitochondrial status, and lipid handling in *Caenorhabditis elegans*. DHM (50–200 μM) extended mean lifespan by 9.1–15.0%, reduced age-related degeneration of ALM (anterior lateral microtubule) and PLM (posterior lateral microtubule) touch-receptor neurons, and preserved locomotor and anterior touch-response performance into later adulthood. Survival after juglone challenge improved with DHM treatment, and the compound showed robust 2,2-diphenyl-1-picrylhydrazyl (DPPH) and 2,2′-azino-bis(3-ethylbenzothiazoline-6-sulfonic acid) (ABTS) radical-scavenging activity. DHM modestly lowered tetramethylrhodamine ethyl ester (TMRE) fluorescence. In a separate cohort assessed under a different exposure schedule, the mtDNA copy number did not differ significantly from that of the vehicle. DHM also increased GFP-negative/mCherry-positive mitochondrial puncta in ALM neurons, patterns consistent with a shifted mitochondrial energetic state and greater mitochondrial trafficking to acidic degradative compartments. The neuroprotective effect persisted in *ucp-4*(ok195) mutants but was not statistically detected in *pink-1*(tm1779); *pdr-1*(gk448) double mutants; since a formal cross-genotype interaction test was not performed, this pattern is treated as hypothesis-generating rather than definitive evidence of pathway dependency. DHM lowered intestinal lipid stores, suppressed *fat-5*, *fat-6*, and *fat-7* expression, and modestly upregulated *atfs-1*. Neither the SOD-3 nor GST-4 canonical reporters showed significant induction, and DHM’s lifespan-extending effect was retained in *daf-16*(mu86) mutants, with an exploratory Cox model providing no evidence of a genotype × dose interaction. Together, these findings indicate that DHM is associated with healthspan-related phenotypes alongside a coordinated pattern of redox regulation, mitochondrial quality-control responses, and metabolic adjustment.

## 1. Introduction

Progressive loss of physiological integrity defines aging, with mitochondrial dysfunction, proteostasis collapse, neurodegeneration, and diminished stress resistance among its most prominent features [1,2]. As central hubs of energy production, redox balance, and cell survival, mitochondria undergo age-related decline that can raise oxidative burden and undermine tissue function. This has given rise to the concept of mitohormesis, in which a mild mitochondrial stressor triggers adaptive protective responses—a framework increasingly used to explain interventions that bolster resilience during aging [3].

Interest in natural dietary compounds, especially polyphenols and flavonoids, as modulators of the aging process has grown substantially, largely because of their combined influence on redox status, mitochondrial function, and stress signaling [4]. Dihydromyricetin (DHM, also called ampelopsin) is a flavanonol (2,3-dihydroflavonol) found abundantly in *Ampelopsis grossedentata* (Tengcha vine) and is reported to have antioxidative, hepatoprotective, anti-inflammatory, and neuroprotective properties [4,5]. Preclinical evidence further points to benefits of DHM for cognitive function, neuroinflammation, and mitochondrial and aging-related phenotypes [6,7,8,9]. In mammalian models of aging, DHM has recently been shown to act as a senomorphic agent—promoting nuclear translocation of peroxiredoxin 2 and reducing tissue aging in prematurely aged mice [10]. Still, exactly how DHM connects antioxidant activity to mitochondrial quality control and metabolic remodeling in a living, aging organism remains poorly defined.

*Caenorhabditis elegans* offers a strong system for addressing this gap: a short lifespan paired with conserved stress-response and metabolic pathways, alongside readily measurable indicators of locomotion, lipid storage, neuronal health, and age-related behavior [11,12,13]. Genetic tools available in this organism allow functional dissection of IIS/FOXO signaling, SKN-1/Nrf2-dependent stress pathways, mitochondrial stress adaptation, and PINK-1/PDR-1-mediated mitochondrial quality control. Tandem green/red mitochondrial reporters take advantage of differential fluorophore stability under acidic conditions, offering a puncta-based readout of mitochondrial trafficking into lysosome-like degradative compartments [14,15]. Together, these features make *C. elegans* well suited for linking whole-organism healthspan phenotypes to underlying mitochondrial and redox mechanisms.

A number of flavonoids have already been shown to promote healthspan in *C. elegans*. Quercetin, for instance, extends lifespan and enhances stress resistance in part by modulating mitochondrial metabolism and reactive oxygen species [16], while resveratrol supports mitochondrial and stress-adaptive responses via conserved nutrient-sensing pathways [17]. Additional *C. elegans* work has shown that natural antioxidants—including quercetin, naringenin, and green-tea compounds—can enhance stress resistance and/or extend lifespan through conserved stress-response signaling [18,19,20]. Our own earlier work demonstrated that modulating mitochondrial energetics can blunt age-associated neurodegeneration in this organism [21,22], and more recently, using a closely related experimental design, we found that morin extends lifespan while preserving mechanosensory neuron integrity, locomotor function, and lipid balance alongside mitochondrial stress adaptation [23]. Building on these findings, we hypothesized that DHM might similarly protect neuronal function and healthspan but through a distinct response profile linking direct antioxidant action to adaptive shifts in mitochondrial energetics, quality control, and metabolic remodeling.

In the present work, we characterized how DHM affects lifespan, age-related mechanosensory neurodegeneration, locomotor function, resistance to oxidative stress, direct radical-scavenging capacity, intestinal lipid accumulation, mitochondrial membrane potential, mitophagy-associated mitochondrial trafficking, stress/metabolic gene expression, and DAF-16/SKN-1 subcellular localization. We additionally employed *daf-16*(mu86), *ucp-4*(ok195), and *pink-1*(tm1779); *pdr-1*(gk448) mutant backgrounds as functional genetic tools to test whether canonical longevity signaling, mitochondrial energetic regulation, or mitochondrial quality-control pathways shape the DHM response. Rather than assuming a single linear mechanism from the outset, this integrated experimental approach was designed to examine whether coordinated redox, mitochondrial, and metabolic adaptations accompany DHM-associated neuroprotection and healthspan-related phenotypes.

## 2. Materials and Methods

### 2.1. Chemicals and Compound Preparation

Dihydromyricetin (DHM; purity > 98%; Sigma-Aldrich, St. Louis, MO, USA) was dissolved in dimethyl sulfoxide (DMSO) as a stock solution. Unless otherwise stated, all other chemicals and reagents, including DMSO, FUdR (5-fluoro-2′-deoxyuridine), juglone, CCCP (carbonyl cyanide *m*-chlorophenyl hydrazone), Orlistat, Oil Red O, TMRE, levamisole, sodium azide, Triton X-100, DPPH, ABTS, potassium persulfate, and ascorbic acid, were purchased from Sigma-Aldrich. Working solutions of 50, 100, and 200 μM DHM were prepared fresh in 0.5% (*v*/*v*) DMSO vehicle for every experimental condition. Media and plates were replaced every 48 h to maintain compound freshness, and exposure to light was minimized throughout. In preliminary control experiments, 0.5% DMSO alone had no detectable effect on lifespan or neuronal morphology in *C. elegans*. The three concentrations tested fall within the range typically used to assess flavonoid bioactivity in this organism [21,22,23]. Where experimental procedures overlapped with our earlier study of morin, the same core workflow was followed; details specific to DHM (concentrations, treatment durations, controls, and statistics) are reported separately below in full.

### 2.2. Worm Strains and Maintenance

Experiments used wild-type N2 (Bristol) *C. elegans* along with the reporter and mutant lines listed below. Age-related neuronal abnormalities were scored using the zdIs5 [P*mec-4*::GFP] line, which labels the ALM (anterior lateral microtubule) and PLM (posterior lateral microtubule) mechanosensory neuron pairs. ALM and PLM were selected because they are well-characterized touch-receptor neurons with a stereotyped, easily scored morphology that is widely used as a tractable readout of age-associated neurodegeneration in *C. elegans*; preservation of these two neuron pairs is not assumed to generalize to neuroprotection across the nervous system as a whole. To visualize mitochondrial trafficking toward acidic degradative compartments in ALM neurons, we generated an in-house extrachromosomal *mec-4*p::COX8::GFP::mCherry tandem reporter, co-injected with *rol-6*(su1006) as a selection marker. To probe pathway involvement, the zdIs5 reporter was crossed into *pink-1*(tm1779); *pdr-1*(gk448) and *ucp-4*(ok195) mutant backgrounds, and homozygosity at each locus was verified by PCR genotyping prior to experimentation. The requirement of DAF-16 for DHM-induced longevity was tested in CF1038 *daf-16*(mu86) mutants. Additional GFP-based reporter strains employed were TJ356 zIs356 IV [*daf-16*p::*daf-16*a/b::GFP + *rol-6*(su1006)], LD1 ldIs7 [*skn-1*b/c::GFP + *rol-6*(su1006)], CF1553 muIs84 [(pAD76) *sod-3*p::GFP + *rol-6*(su1006)], and CL2166 dvIs19 III [*gst-4*p::GFP::NLS]. The *pink-1*(tm1779) mutant was provided by the National BioResource Project (NBRP; Tokyo, Japan). All other strains were obtained from the Caenorhabditis Genetics Center (CGC; University of Minnesota, Minneapolis, MN, USA). All strains were kept at 20 °C, either on NGM agar seeded with freeze-dried *E. coli* HB101 (BioVirid, Veenendaal, The Netherlands) or in liquid S-complete medium using the same food source, consistent with our established culturing approach [23,24]. For liquid-culture assays, a 10× HB101 stock was diluted 1:10 (*v*/*v*) in S-complete medium immediately before use. Depending on the population size each assay required, synchronized cohorts were generated either by a 6 h timed egg lay or by alkaline hypochlorite bleaching followed by L1 arrest and subsequent regrowth [23].

### 2.3. Lifespan Assay

Survival was measured in liquid culture following a protocol adapted from our prior work [23]. Synchronized L4-stage larvae (approximately 10 animals per well) were dispensed into 96-well plates containing S-complete medium, *E. coli* HB101 at the working concentration, 50 μM FUdR to block progeny production, and either DHM (50, 100, or 200 μM in 0.5% DMSO) or vehicle alone. Plates were held at 20 °C under gentle agitation (150 rpm), and survival was checked once daily; an animal was scored dead if it no longer responded to a gentle mechanical tap and showed no pharyngeal pumping. Worms that died from internal hatching, ruptured due to extruded organs, or were lost/desiccated along the well wall were censored at their last observed time point. Kaplan–Meier curves were plotted, and survival differences were tested using the log-rank (Mantel–Cox) method in GraphPad Prism 10 (GraphPad Software, San Diego, CA, USA). Each treatment arm was run in three independent biological replicates; full survival data appear in Supplementary Table S1.

### 2.4. Neurodegeneration Assay

Neuronal integrity across adulthood was assessed in zdIs5 [P*mec-4*::GFP] animals using the same general approach as our previous morin study [23], adjusted for DHM-specific dosing. Synchronized L4 worms were exposed to vehicle or DHM (50, 100, or 200 μM) in liquid S-complete medium for 3 days, then moved to HB101-seeded NGM plates carrying the matching treatment, with media refreshed every 48 h. On days 5, 10, and 15 of adulthood, worms were mounted on agar pads, anesthetized with 20 mM sodium azide, and imaged on an Olympus FV1000 confocal laser-scanning microscope (Olympus, Tokyo, Japan) at the Core Facility for Supporting Analysis & Imaging of Biomedical Materials, Wonkwang University (supported by the National Research Facilities and Equipment Center, NFEC-2021–08–272253). A minimum of 120 ALM and 120 PLM neurons was scored per condition, pooled across three independent replicates. Structural defects—soma outgrowths, axonal branching, wavy axons, neurite tangling, and axon blebbing—were classified according to previously established criteria for age-related touch-neuron morphology [25,26]. Identical scoring was applied to zdIs5; *pink-1*(tm1779); *pdr-1*(gk448) and zdIs5; *ucp-4*(ok195) animals under vehicle or DHM treatment. All scoring was done blind to treatment condition. Effects of age and treatment were tested by two-way ANOVA with Dunnett’s multiple-comparisons test, performed separately within each genetic background; because this analysis was not a factorial cross-genotype test, a treatment effect present in one background but absent in another was not taken as formal evidence of a genotype-by-treatment interaction. Full statistics appear in Supplementary Table S2.

### 2.5. Locomotion Assay

Locomotor activity and mechanosensory function were assessed at adult days 3, 5, 7, and 9. Measurements were not extended beyond day 9 because we observed a floor effect for *C. elegans* locomotory function approaching day 15 of adulthood in our preliminary investigations, limiting discrimination among treatment groups at that age; age-related loss of movement can also complicate interpretation of touch-evoked reversals [27,28]. We therefore restricted quantitative comparisons to the day 3–9 window, during which the majority of animals retain normal locomotory capacity, and paired the touch-response assay with locomotion measurements at identical time points and DHM concentrations (50, 100, and 200 μM) to evaluate these outcomes at comparable ages. Locomotor activity was measured using the WMicrotracker SMART system (Phylumtech, Santa Fe, Argentina), following our previously published workflow [23]. Synchronized adult populations (≥30 animals per plate) were placed on food-free 35 mm NGM plates at 20 °C, allowed to acclimate for 30 min, and recorded for 5 min under identical acquisition settings; the system uses infrared imaging and automated path tracking to derive movement parameters, including mean speed. Each condition/timepoint combination was evaluated in three independent biological replicates. Data were analyzed by two-way ANOVA (age × treatment) with Dunnett’s multiple-comparisons test comparing each DHM concentration to vehicle within each age; statistics are in Supplementary Table S3. Anterior touch responsiveness—mediated by both ALM and AVM mechanosensory neurons and therefore not specific to ALM [29,30]—was evaluated at the same adult timepoints and DHM concentrations. Age-synchronized animals were transferred to a fresh, unseeded NGM plate and allowed to acclimate for 5 min. Touch responsiveness was scored by stroking the animal immediately behind the pharynx with an eyebrow hair; each animal received 10 repetitive anterior touches with a minimum 10 s interval between touches to minimize habituation, and the percentage of touches eliciting a reversal response was recorded as percent responsiveness. At least 30 animals per group were scored in each of three independent biological replicates. Data were analyzed by two-way ANOVA (age × treatment) with Dunnett’s multiple-comparisons test comparing each DHM concentration to vehicle within each age; statistics are in Supplementary Table S4. Posterior (PLM-mediated) touch responsiveness was not assessed in this study.

### 2.6. Juglone-Induced Oxidative Stress Resistance Assay

In vivo antioxidant capacity was assessed using an acute juglone (5-hydroxy-1,4-naphthoquinone; Sigma-Aldrich) challenge, a superoxide-generating redox cycler routinely applied in *C. elegans* stress-resistance studies [31]. A 50 mM juglone stock was made in DMSO, stored at −20 °C protected from light, and diluted fresh before each experiment. Day-1 adult N2 animals were pre-exposed to DHM (50, 100, or 200 μM) or vehicle (0.5% DMSO) for 24 h under normal culture conditions, then rinsed three times in M9 buffer before being transferred to liquid M9 containing 300 μM juglone for a 2 h exposure at 20 °C in the dark. Immediately afterward, survival was scored by gentle prodding with a platinum wire; animals lacking a response and pharyngeal pumping were counted as dead. At least 100 worms per group were assessed across three independent biological replicates. Group differences were evaluated by one-way ANOVA with Dunnett’s multiple-comparisons test against the juglone-exposed vehicle group.

### 2.7. GFP Reporter and Localization Assays

Transcription-factor localization and downstream reporter output were examined using TJ356 (DAF-16::GFP), LD1 (SKN-1::GFP), CF1553 (*sod-3*p::GFP), and CL2166 (*gst-4*p::GFP) strains. Synchronized L4 animals received vehicle or DHM (50, 100, or 200 μM); DAF-16::GFP, *sod-3*p::GFP, and *gst-4*p::GFP were scored 24 h later, while SKN-1::GFP was evaluated after 14 h. Animals were mounted on 2% agarose pads and immobilized with 1 mM levamisole before imaging on a Nikon Eclipse 80i microscope (Nikon, Tokyo, Japan) fitted with a DS-Fi3 camera (Nikon), using consistent exposure/gain settings within each reporter assay, following the imaging approach used previously for DAF-16::GFP [23]. DAF-16::GFP and SKN-1::GFP localization were each classified by a blinded observer into cytosolic, intermediate, or nuclear categories; at least 30 animals per group were scored across three biological replicates (range 30–42 per replicate), and results are summarized as replicate-level percentages. Because the three localization categories are mutually exclusive and sum to 100%, we treat the two-way ANOVA (concentration × category) with Dunnett comparisons as an exploratory descriptive analysis rather than a formal test of the full compositional distribution; these data were additionally analyzed using an ordinal logistic regression (proportional-odds model), with localization category as the ordinal outcome and DHM concentration as a continuous predictor, accompanied by a linear-by-linear (Cochran–Armitage-type) trend test on the same pooled contingency table; neither pooled test adjusted for biological replicate clustering, and the proportional-odds assumption was not formally verified. For *sod-3*p::GFP and *gst-4*p::GFP, whole-body fluorescence intensity was measured in ImageJ (version 1.54r; National Institutes of Health, Bethesda, MD, USA) and normalized to body area, with one-way ANOVA/Dunnett’s test used to compare each treatment against vehicle. At least 30 animals per group were scored in each of three independent replicates.

### 2.8. Tandem Mitochondrial Reporter Assay

A puncta-based readout consistent with mitochondrial delivery to acidic degradative compartments was evaluated in ALM neurons with the *mec-4*p::COX8::GFP::mCherry tandem reporter. Because GFP is more acid-sensitive than mCherry, mitochondria that reach acidic lysosomal compartments lose GFP signal while retaining mCherry [14,15]; GFP-negative/mCherry-positive puncta were therefore scored as mitolysosome-associated structures. Synchronized L4 animals were exposed to vehicle or DHM (50, 100, or 200 μM) for 3 days, alongside a 15 μM CCCP positive-control arm over the same interval. On day 3 of adulthood, animals were imaged on a Nikon Eclipse 80i fluorescence microscope with a DS-Fi3 camera under fixed dual-channel acquisition settings (GFP: 488 nm excitation/500–530 nm emission; mCherry: 543 nm excitation/590–640 nm emission). Puncta were counted only within ALM cell bodies; the full ALM axon could not be consistently traced in live animals, and head-region imaging was complicated by the rolling phenotype conferred by the *rol-6*(su1006) co-injection marker. PLM neurons were not scored in this assay. Because this reporter is carried on an extrachromosomal array, animals can vary in array copy number and show mosaic silencing; only animals with clearly detectable fluorescence in both channels were included, and all images were acquired under identical, fixed settings across treatment groups, but residual array-related expression variability cannot be fully excluded. Fifty ALM neurons were scored per group in each of three biological replicates (150 neurons per treatment overall). Comparisons used one-way ANOVA with Dunnett’s multiple-comparisons test against vehicle, treating the biological replicate mean as the unit of analysis. Statistics are reported in Supplementary Table S5.

### 2.9. Oil Red O Staining

Intestinal lipid content was measured by Oil Red O staining following the O’Rourke et al. protocol [32] as previously implemented in our lab [23]. Synchronized L4 larvae were cultured for 3 days under one of the following conditions: vehicle (0.5% DMSO), 12 μM Orlistat, DHM alone (50, 100, or 200 μM), vehicle plus 2% glucose (high glucose, HG), 12 μM Orlistat plus 2% glucose, or DHM (50, 100, or 200 μM) plus 2% glucose; all conditions contained 0.5% DMSO. After treatment, animals were washed in M9 buffer, fixed in 60% isopropanol, and stained for 2 h in freshly made Oil Red O solution (0.5% Oil Red O in isopropanol, diluted 3:2 with water). Unbound dye was removed with PBS containing 0.01% Triton X-100. Worms were mounted on 2% agarose pads with 10 mM sodium azide and imaged under DIC optics on a Nikon Eclipse 80i microscope. At least 70 animals per group were quantified in ImageJ. Comparisons used one-way ANOVA with Dunnett’s multiple-comparisons test, referenced against vehicle for basal conditions or against HG + vehicle for high-glucose conditions. Data are provided in Supplementary Table S6.

### 2.10. TMRE Staining

Mitochondrial membrane potential (ΔΨ_m_) was measured with TMRE (tetramethylrhodamine ethyl ester; Sigma-Aldrich), adapting our previously established protocol [23,33]. Synchronized L4 animals were exposed for 6 h to DHM (50, 100, or 200 μM), 50 μM CCCP, or vehicle, after which TMRE was added directly to the culture at a final concentration of 100 nM (treatment compound not removed) for a further 4 h incubation in the dark. Animals were then moved to HB101-seeded plates for 2 h to wash out unincorporated dye, immobilized, and imaged on a Nikon Eclipse 80i microscope with a DS-Fi3 camera under matched exposure/gain settings. Whole-body TMRE signal was quantified in ImageJ and normalized to body area, with at least 50 animals scored per group across three biological replicates. Comparisons used one-way ANOVA with Dunnett’s multiple-comparisons test versus vehicle; statistics are in Supplementary Table S7.

As an indirect, separately timed assessment of mitochondrial content, the relative mtDNA copy number was quantified by qPCR. One-day-old adults were treated with 100 μM DHM or 0.5% DMSO for 48 h, under a different developmental-stage and exposure schedule from TMRE staining, in five independent biological replicates. The mtDNA copy number was determined by qPCR targeting the mitochondrially encoded gene *nduo-1* (ND1; forward 5′-AGCGTCATTTATTGGGAAGAAGAC-3′, reverse 5′-AAGCTTGTGCTAATCCCATAAATGT-3′), normalized to the single-copy nuclear-encoded gene *cox-4* (forward 5′-GCCGACTGGAAGAACTTGTC-3′, reverse 5′-GCGGAGATCACCTTCCAGTA-3′), following an established real-time PCR-based approach for *C. elegans* mtDNA quantification [34], and calculated using the 2^−ΔΔCT^ method. Data are in Supplementary Table S12.

### 2.11. Real-Time PCR (RT-qPCR)

Gene expression changes were assessed using an RT-qPCR pipeline adapted from our morin study [23]. One-day-old adults were exposed to 100 μM DHM or 0.5% DMSO for 48 h, with five independent biological replicates per condition. Animals were washed off plates with M9 buffer and allowed to settle by gravity; roughly 50 μL of packed worm pellet per replicate was used for RNA extraction with the RNeasy Mini Kit (Qiagen, Germantown, MD, USA). First-strand cDNA was synthesized from 1 μg total RNA with the High-Capacity cDNA Reverse Transcription Kit (Applied Biosystems, Foster City, CA, USA). qPCR reactions used PowerUp SYBR Green Master Mix on a QuantStudio 1 instrument (Applied Biosystems), with cycling at 95 °C for 10 min followed by 40 cycles of 95 °C/15 s and 60 °C/30 s, including melt-curve analysis. Expression levels were normalized to *cdc-42* [35,36], and fold change was calculated by the 2^−ΔΔCT^ method [37]. The gene panel spanned IIS/FOXO and longevity regulators (*aak-2*, *daf-2*, *daf-16*, *skn-1*, *sir-2.1*); mitochondrial stress-adaptation genes (*atfs-1*, *hsp-6*, *hsp-60*, *polg-1*, *fmo-2*); mitophagy genes (*pink-1*, *pdr-1*); antioxidant/stress-defense genes (*hsf-1*, *hsp-16.2*, *gst-4*, *ctl-1*, *sod-3*); and lipid-metabolism genes (*acs-2*, *nhr-49*, *fat-5*, *fat-6*, *fat-7*). Statistical comparisons versus vehicle (Šidák-adjusted) were run on ΔCT values, while fold-change is reported as 2^−ΔΔCT^. The 100 μM dose was chosen for this panel because it produced consistent effects across the phenotypic assays. Primer sequences are listed in Supplementary Table S8, with full results and statistics in Supplementary Table S9.

### 2.12. DPPH and ABTS Radical-Scavenging Assays

The intrinsic radical-scavenging capacity of DHM was measured in vitro with the DPPH (2,2-diphenyl-1-picrylhydrazyl) and ABTS (2,2′-azino-bis(3-ethylbenzothiazoline-6-sulfonic acid)) assays. For DPPH, following a modified version of the Malterud et al. method [38], a 0.3 mM DPPH solution was made in methanol, protected from light, and allowed to equilibrate in the dark at room temperature for 30 min. DHM stocks were prepared so that 1:1 mixing with the DPPH reagent gave final well concentrations of 25, 50, 100, and 200 μM; ascorbic acid was included at a single reference concentration of 250 μg/mL (~1.42 mM). In a 96-well plate, 100 μL of DHM or ascorbic-acid solution was combined with 100 μL of 0.3 mM DPPH (final concentration 0.15 mM) and incubated in the dark at room temperature for 30 min before absorbance was read at 517 nm using a microplate reader (Molecular Devices, San Jose, CA, USA). DPPH scavenging (%) was calculated as {1 − (Abs_sample − Abs_blank)/Abs_control} × 100, using solvent-matched blank and control wells. For ABTS, following a modified Re et al. protocol [39], the ABTS•+ radical cation was generated by combining 7.4 mM ABTS with 2.6 mM potassium persulfate (1:1 *v*/*v*) and incubating in the dark at room temperature for 24 h; the resulting stock was diluted in PBS to an absorbance of 0.6–0.7 at 734 nm before use. In a 96-well plate, 100 μL of DHM or ascorbic-acid solution was mixed with 100 μL of working ABTS•+ solution (1:1), matching the same final concentrations as above, and absorbance was read at 734 nm using the same microplate reader after 30 min in the dark. ABTS scavenging (%) was calculated as (Abs_blank − Abs_sample)/Abs_blank × 100, relative to a vehicle-matched solvent control. Each assay was run in three independent replicates. Because the ascorbic-acid reference was tested at only one concentration, these assays establish DHM’s intrinsic scavenging capacity rather than its relative molar potency against ascorbic acid.

### 2.13. Statistical Analysis

Unless stated otherwise, data are shown as mean ± SEM, with significance set at *p* < 0.05. Two-group comparisons used unpaired two-tailed Student’s *t*-tests where appropriate, and multi-group comparisons used one-way or two-way ANOVA followed by Dunnett’s multiple-comparisons test versus vehicle, as specified per experiment. Survival data were analyzed with Kaplan–Meier curves and log-rank (Mantel–Cox) tests. For assays scoring multiple animals or neurons within a single experimental run (neurodegeneration, locomotion, Oil Red O, TMRE, tandem-reporter puncta, and juglone survival), the biological replicate mean—not the individual animal or neuron—served as the unit of statistical analysis (*n* = 3 unless otherwise noted); the number of animals/neurons scored per replicate (*N*) is reported alongside each result to indicate scoring depth and is distinct from the statistical sample size. Localization-category analyses are explicitly flagged as exploratory, since the cytosolic/intermediate/nuclear percentages are compositional and non-independent, and were supplemented with an ordinal logistic regression and a pooled linear-by-linear trend test. Neurodegeneration analyses in mutant backgrounds were conducted separately within each genotype; accordingly, a non-significant result in a mutant background was not interpreted as evidence of a significant genotype-by-treatment interaction. All neuronal and imaging scoring was performed blind to treatment. Unless otherwise specified, experiments comprised at least three independent biological replicates. For the targeted RT-qPCR panel, the biological replicate was the unit of analysis (*n* = 5), with Šidák-adjusted *p*-values reported across the 22-gene panel in Table S9.

To formally test whether DAF-16 modifies the DHM lifespan response, individual-level survival data from wild-type and *daf-16*(mu86) animals (Section 2.3) were combined and analyzed using a Cox proportional hazards model, with genotype, DHM concentration (continuous, 0–200 μM), and their interaction as covariates; the proportional-hazards assumption was not formally tested, and the model did not adjust for clustering by biological replicate. A non-significant genotype × concentration interaction term indicates that this analysis did not detect evidence of genotype-dependent modification of the DHM dose–response relationship, rather than confirming its absence. The Cox proportional hazards model and the ordinal logistic regression were performed in Python 3.14.7 using the lifelines and statsmodels packages, respectively.

## 3. Results

### 3.1. DHM Extends Lifespan in C. elegans

**Figure 1:**
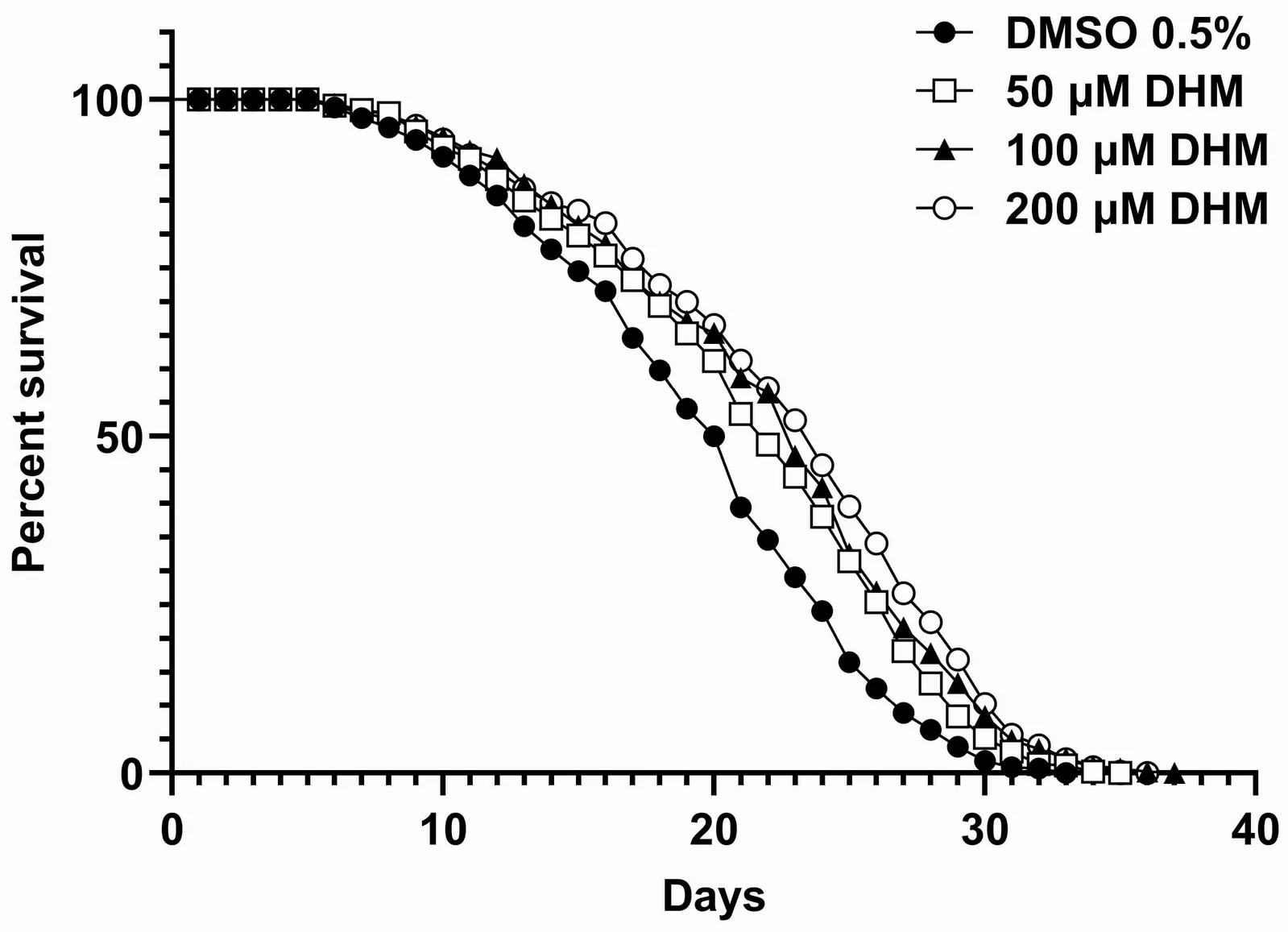
Kaplan–Meier survival curves of *C. elegans* treated with dihydromyricetin (DHM). Age-synchronized worms were treated with vehicle control (0.5% dimethyl sulfoxide, DMSO) or DHM at 50, 100, or 200 μM. Survival was monitored daily until all worms had died. Curves represent pooled data from three independent assays. Statistical analysis was performed by log-rank (Mantel–Cox) tests.

Three independent lifespan assays demonstrated a reproducible extension of survival in DHM-treated worms compared with the vehicle control (0.5% DMSO) (Figure 1; Table 1). Vehicle-treated animals exhibited a mean lifespan of 19.96 ± 0.31 days, a median lifespan of 20.5 days, and a maximum lifespan of 33 days. DHM increased mean lifespan to 21.78 ± 0.32, 22.45 ± 0.33, and 22.96 ± 0.34 days at 50, 100, and 200 μM, respectively, corresponding to extensions of approximately 9.1%, 12.5%, and 15.0%. Median lifespan increased to 22, 23, and 24 days, respectively. Log-rank analysis confirmed significant survival benefits at all three concentrations (50 μM, 100 μM, and 200 μM, each *p* < 0.0001). These data establish a dose-responsive pro-longevity effect of DHM in wild-type *C. elegans*.

### 3.2. DHM Attenuates Age-Associated Mechanosensory Neurodegeneration

**Figure 2:**
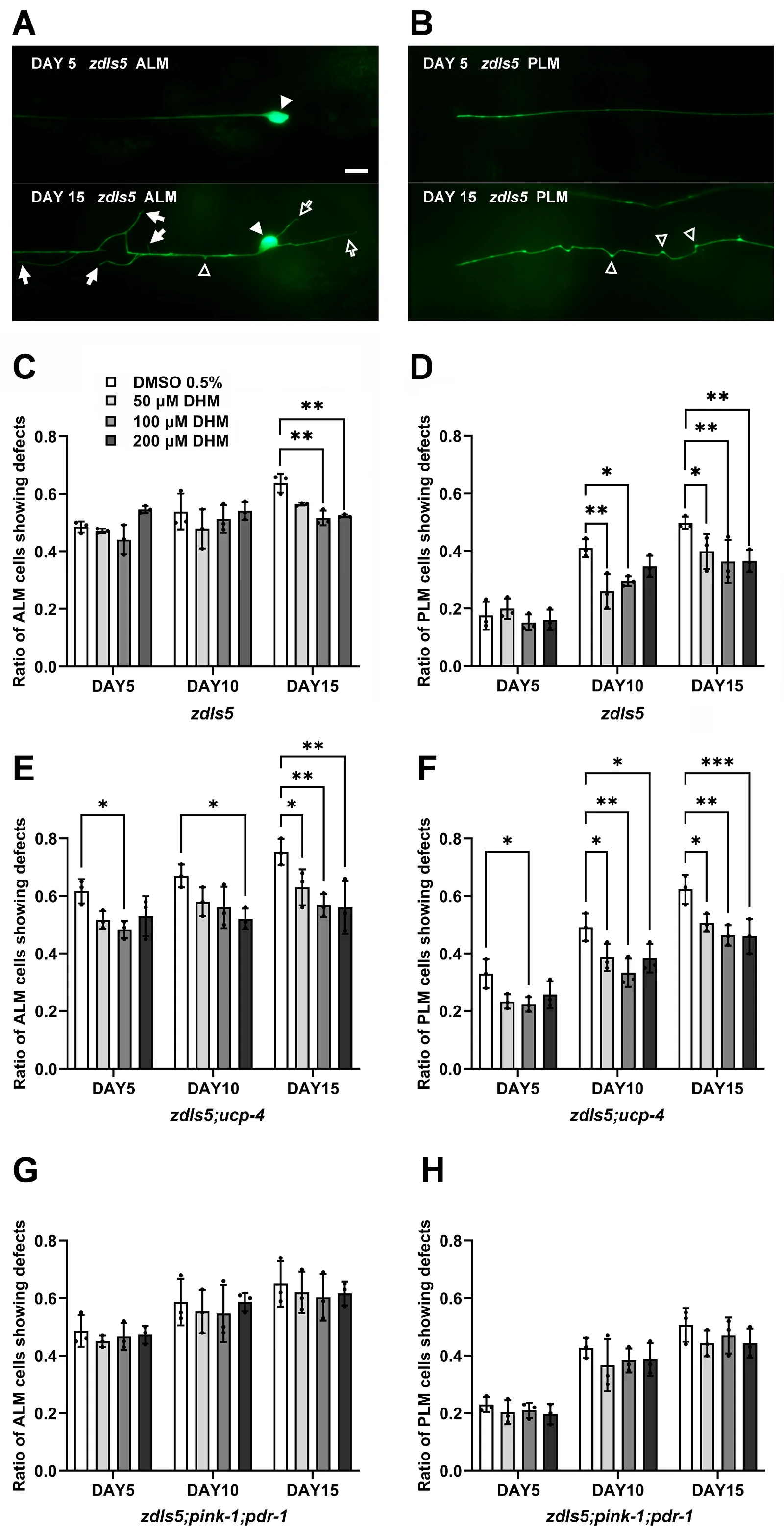
Dihydromyricetin attenuates age-related mechanosensory neuronal degeneration and shows differential efficacy across mitochondrial regulatory mutant backgrounds. (**A**,**B**) Representative fluorescence images of zdIs5 [P*mec-4*::GFP] ALM (**A**) and PLM (**B**) neurons at adult days 5 and 15, illustrating the morphological criteria used for defect scoring; filled arrowheads indicate the ALM cell body, open arrowheads indicate axon blebs, and arrows indicate ectopic branching. Scale bar = 10 μm. (**C**,**D**) Quantification of ALM (**C**) and PLM (**D**) neuronal defects in zdIs5 worms at adult days 5, 10, and 15 following vehicle or DHM (50, 100, or 200 μM) treatment. (**E**,**F**) ALM and PLM neuronal defects in zdIs5; *ucp-4*(ok195) animals treated with vehicle or DHM. (**G**,**H**) ALM and PLM neuronal defects in zdIs5; *pink-1*(tm1779); *pdr-1*(gk448) animals treated with vehicle or DHM. DHM did not significantly suppress neuronal degeneration in the double-mutant background at the examined concentrations and timepoints. Because analyses were performed separately within each genotype, the difference in statistical significance across backgrounds is treated as hypothesis-generating and does not establish an exclusive linear pathway. Data represent mean ± SEM from three independent biological replicates. Circles indicate each replicate mean. * *p* < 0.05, ** *p* < 0.01, *** *p* < 0.001.

Neuronal integrity was examined using zdIs5 [P*mec-4*::GFP] worms, which label ALM and PLM mechanosensory neurons (Figure 2A–D). Vehicle-treated animals showed progressive age-associated structural abnormalities, including neurite branching, axonal waviness, tangling, blebs, and soma-associated outgrowths. DHM attenuated these abnormalities most clearly at later ages. In ALM neurons, 100 and 200 μM DHM reduced the proportion of degenerating neurons at adult day 15 by approximately 18.9% and 17.9%, respectively (both *p* < 0.01). In PLM neurons, protection emerged by day 10 and remained evident at day 15; all three DHM concentrations significantly reduced PLM defects at day 15 by approximately 20.0–27.1%. These results show reduced age-associated morphological abnormalities in the ALM and PLM mechanosensory neurons examined. The behavior of this phenotype in mitochondrial regulatory mutant backgrounds was evaluated separately after characterization of the mitochondrial response (Section 3.8).

### 3.3. DHM Preserves Locomotor Function During Aging

**Figure 3:**
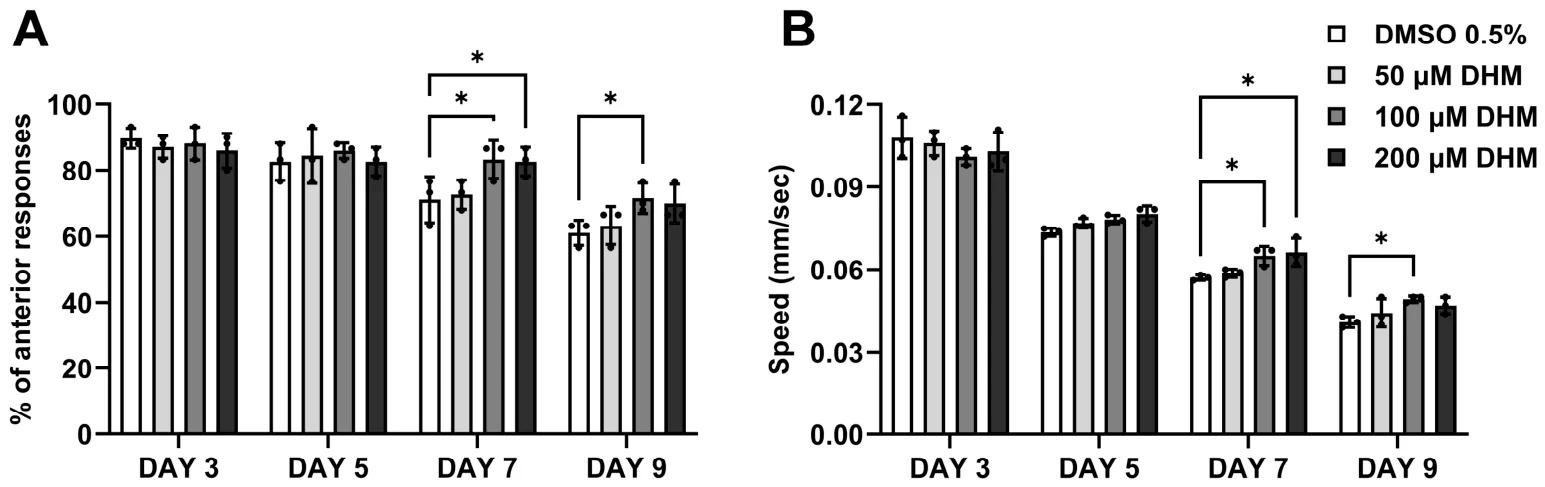
Dihydromyricetin preserves locomotor activity and anterior touch responsiveness during aging. (**A**) Anterior touch responsiveness (reflecting ALM and AVM function) measured at adult days 3, 5, 7, and 9 (30 worms per replicate, three independent biological replicates per condition). Two-way ANOVA showed significant age (*p* < 0.0001) and treatment (*p* = 0.0200) effects, without a significant age × treatment interaction (*p* = 0.1984); Dunnett’s test identified significant increases at day 7 (100 μM, adjusted *p* = 0.0177; 200 μM, adjusted *p* = 0.0259) and day 9 (100 μM, adjusted *p* = 0.0477). Posterior (PLM-mediated) touch responsiveness was not assessed. (**B**) Locomotor speed measured using the WMicrotracker SMART system at the same timepoints and concentrations. Two-way ANOVA showed a significant age × treatment interaction (*p* = 0.0264); Dunnett’s multiple-comparisons test versus vehicle within each age identified significant increases at day 7 with 100 μM (adjusted *p* = 0.0404) and 200 μM DHM (adjusted *p* = 0.0146), and at day 9 with 100 μM DHM (adjusted *p* = 0.0316). Data represent mean ± SEM from three independent biological replicates. Circles indicate each replicate mean. * *p* < 0.05.

Locomotor activity was measured at adult days 3, 5, 7, and 9 using the WMicrotracker SMART system (≥30 worms per replicate; Figure 3B). Two-way ANOVA showed a significant age × treatment interaction (*F*(9,32) = 2.516, *p* = 0.0264), a strong age effect (*F*(3,32) = 517.9, *p* < 0.0001), and a treatment effect (*F*(3,32) = 3.016, *p* = 0.0442). No significant treatment differences were detected at days 3 or 5. At day 7, vehicle-treated worms showed a mean speed of 0.0570 mm/s, compared with 0.0650 mm/s at 100 μM DHM (adjusted *p* = 0.0404) and 0.0663 mm/s at 200 μM DHM (adjusted *p* = 0.0146); 50 μM DHM was not significant. At day 9, 100 μM DHM increased mean speed from 0.0410 to 0.0493 mm/s (adjusted *p* = 0.0316), whereas the 50 and 200 μM groups were not significant. These results indicate that DHM preserves locomotor performance selectively at later adult ages.

To determine whether this preserved locomotor performance reflects intact mechanosensory neuron function, anterior touch responsiveness (reflecting ALM and AVM function) was evaluated at the same timepoints and concentrations (30 worms per replicate, three independent biological replicates per condition; Figure 3A). Two-way ANOVA revealed a strong age effect (*F*(3,32) = 39.31, *p* < 0.0001) and a significant treatment effect (*F*(3,32) = 3.774, *p* = 0.0200), without a significant age × treatment interaction (*F*(9,32) = 1.478, *p* = 0.1984). No significant treatment differences were detected at days 3 or 5. At day 7, vehicle-treated worms showed 71.1% anterior responsiveness, compared with 83.3% at 100 μM DHM (adjusted *p* = 0.0177) and 82.7% at 200 μM DHM (adjusted *p* = 0.0259); 50 μM DHM was not significant (72.7%, adjusted *p* = 0.9665). At day 9, 100 μM DHM increased anterior responsiveness from 61.1% to 71.6% (adjusted *p* = 0.0477), whereas the 50 μM (63.3%, adjusted *p* = 0.9127) and 200 μM (70.0%, adjusted *p* = 0.1055) groups were not significant. These results indicate that DHM preserves anterior mechanosensory function selectively at later adult ages, mirroring the temporal pattern observed for locomotor preservation and providing direct functional support for the neuroprotective phenotype inferred from ALM morphological preservation (Section 3.2). Because posterior touch responsiveness was not assessed, whether PLM function is similarly preserved remains to be determined.

### 3.4. DHM Enhances Resistance to Acute Oxidative Stress

**Figure 4:**
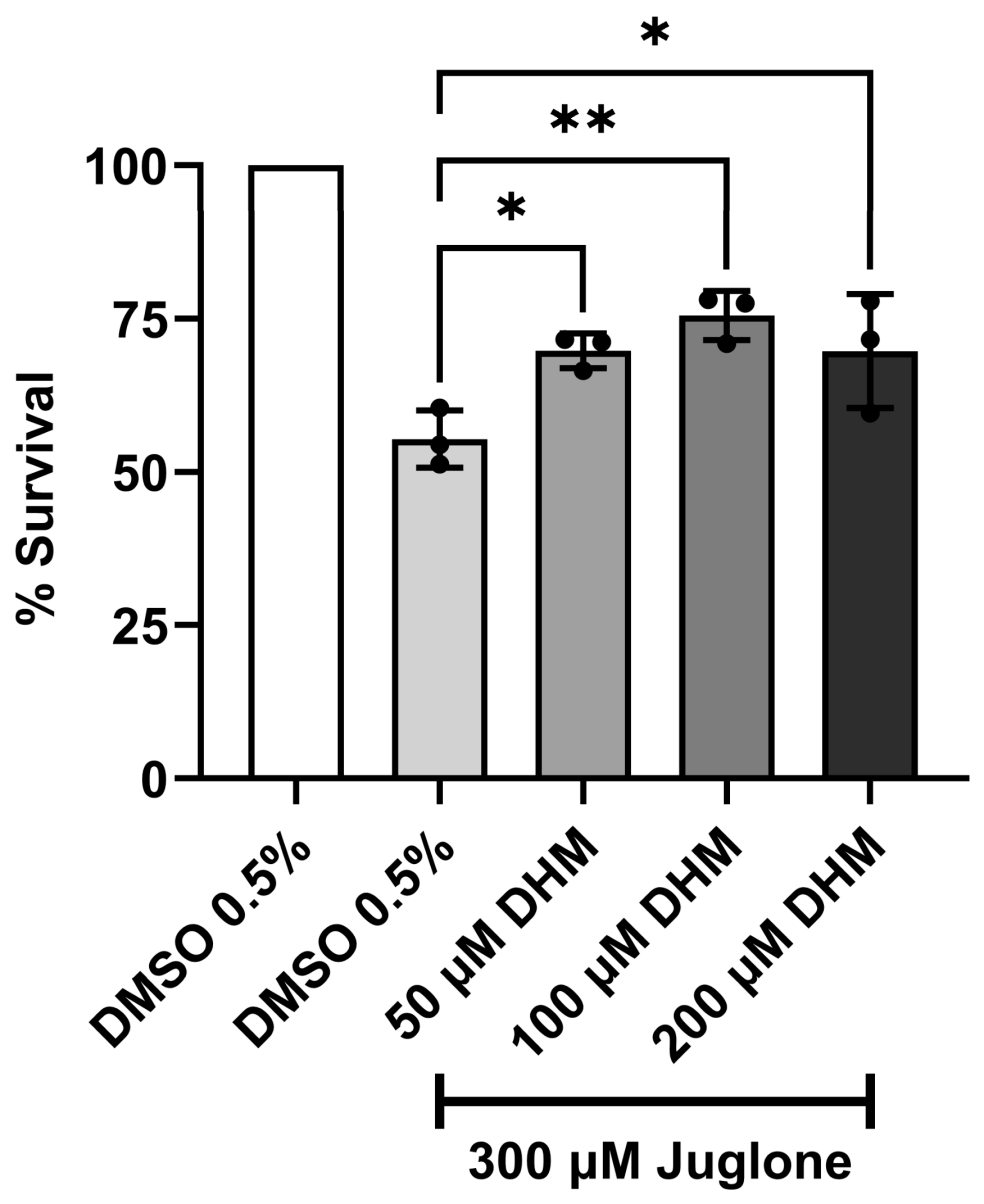
DHM enhances survival of *C. elegans* under juglone-induced oxidative stress. Day-1 adult wild-type (N2) worms were pretreated with DHM (50, 100, or 200 μM) or vehicle (0.5% DMSO) for 24 h, then exposed to 300 μM juglone in liquid M9 medium for 2 h at 20 °C. Survival was assessed immediately by touch response (≥100 worms per group in each of three independent biological replicates). Data represent mean ± SEM. Circles indicate each replicate mean. The first bar shows vehicle-treated animals without juglone exposure. Statistical analysis was performed by one-way ANOVA with Dunnett’s multiple-comparisons test versus the juglone-exposed vehicle group. * *p* < 0.05, ** *p* < 0.01.

To determine whether DHM improves organismal resistance to an acute oxidative challenge, day-1 adult N2 worms were pretreated with DHM for 24 h and subsequently exposed to 300 μM juglone for 2 h (Figure 4). Vehicle-treated worms showed 55.4% survival, whereas DHM pretreatment increased survival to 69.8% at 50 μM (*p* < 0.05), 75.6% at 100 μM (*p* < 0.01), and 69.7% at 200 μM (*p* < 0.05). Although the response was not strictly monotonic, all tested concentrations conferred significant protection. These findings demonstrate that DHM increases resistance to acute redox stress in vivo.

### 3.5. DHM Exhibits Strong Direct Radical-Scavenging Activity In Vitro

**Figure 5:**
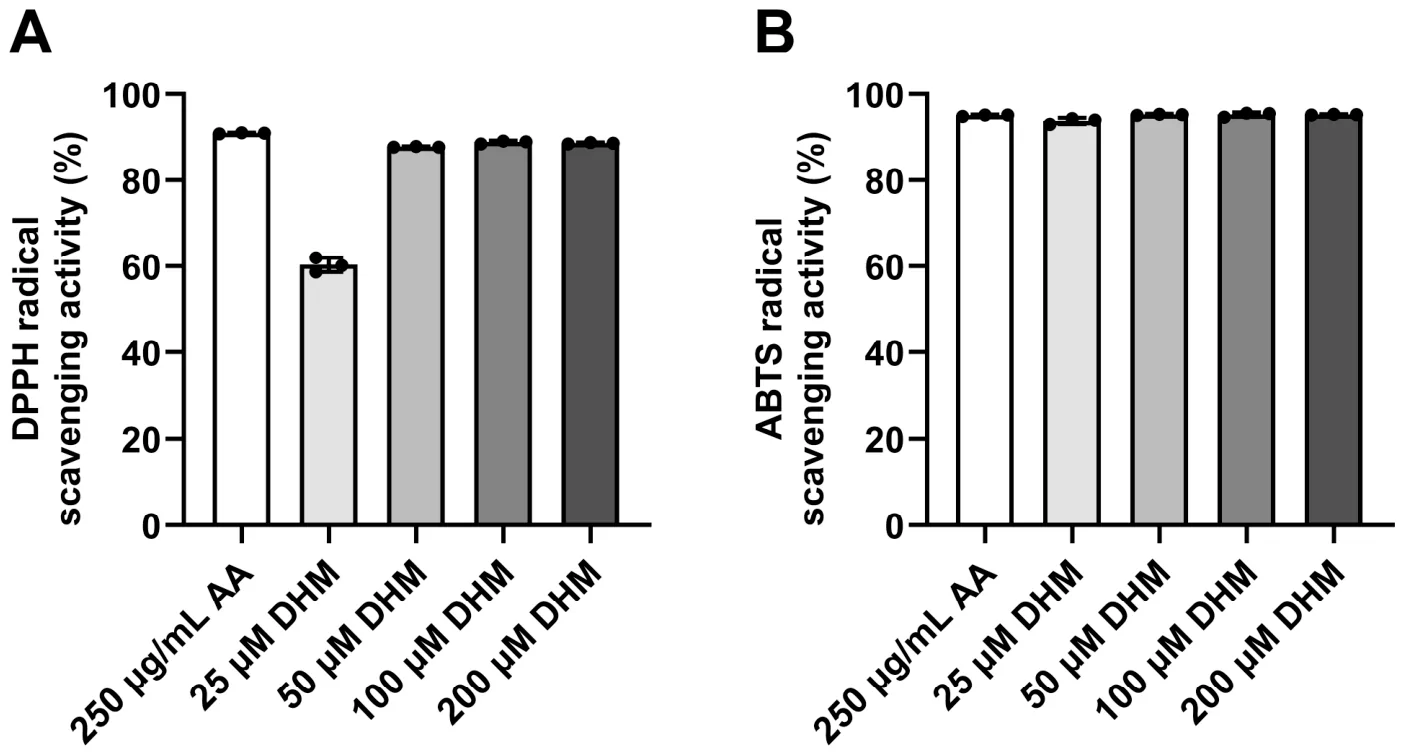
DHM exhibits strong direct radical-scavenging activity in vitro. (**A**) DPPH radical-scavenging activity at final DHM concentrations of 25–200 μM. (**B**) ABTS radical-scavenging activity at final DHM concentrations of 25–200 μM. Ascorbic acid (AA; final concentration 250 μg/mL, approximately 1.42 mM) was included as a single-point reference antioxidant. Because ascorbic acid was not tested as a matched concentration-response series, the figure does not establish relative molar potency between DHM and ascorbic acid. Data are presented as mean ± SEM (*n* = 3 independent assays). Circles indicate each replicate mean.

To evaluate whether DHM also possesses intrinsic, enzyme-independent antioxidant activity, DPPH and ABTS radical-scavenging assays were performed across 25–200 μM DHM, with ascorbic acid (250 μg/mL, approximately 1.42 mM) included as a single-point reference antioxidant (Figure 5). DHM scavenged DPPH radicals in a concentration-dependent manner, increasing from approximately 60% at 25 μM to approximately 87–89% at 50–200 μM. In the ABTS assay, DHM showed consistently high scavenging activity of approximately 94–96% across the tested range. These values approached those obtained with the single-point ascorbic acid reference (~91% for DPPH and ~95% for ABTS). Because ascorbic acid was not tested as a matched concentration-response series, the data do not establish relative molar potency. They nevertheless demonstrate substantial direct radical-scavenging capacity under the assay conditions used.

### 3.6. DHM Modestly Alters Mitochondrial Energetic State

**Figure 6:**
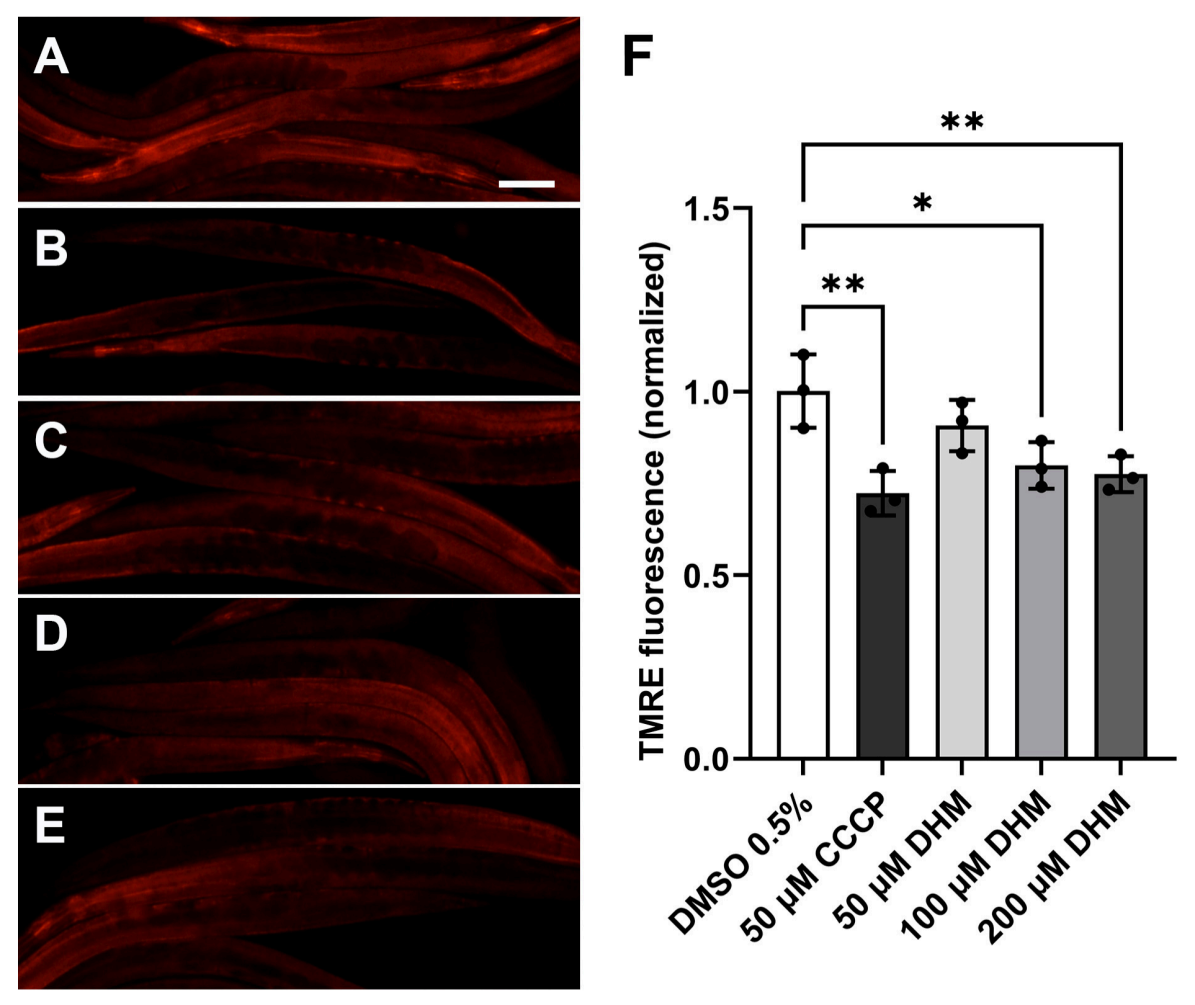
Dihydromyricetin produces a modest reduction in TMRE fluorescence in *C. elegans*. (**A**–**E**) Representative fluorescence micrographs of TMRE-stained worms treated with vehicle (0.5% DMSO), 50 μM CCCP, or DHM at 50, 100, or 200 μM. Scale bar = 100 μm. (**F**) Quantification of whole-body TMRE fluorescence normalized to worm area. CCCP reduced TMRE fluorescence by approximately 27.8%, whereas DHM produced reductions of approximately 9.4% (not significant), 20.2%, and 22.6% at 50, 100, and 200 μM, respectively. Data represent mean ± SEM from three independent biological replicates. Circles indicate each replicate mean. Statistical analysis was performed using one-way ANOVA followed by Dunnett’s multiple-comparisons test versus vehicle. * *p* < 0.05, ** *p* < 0.01.

To determine whether DHM’s antioxidant activity extends beyond direct radical scavenging into effects on mitochondrial physiology, mitochondrial membrane potential (ΔΨm) was assessed by TMRE staining (Figure 6). Relative to vehicle-treated worms (1.002), CCCP (50 μM) reduced TMRE fluorescence by approximately 27.8% (0.724; *p* = 0.0023). DHM produced reductions of approximately 9.4% at 50 μM (0.908; not significant, *p* = 0.3473), 20.2% at 100 μM (0.800; *p* = 0.0180), and 22.6% at 200 μM (0.776; *p* = 0.0093). These data indicate a modest, concentration-associated alteration in mitochondrial energetic state. Because TMRE fluorescence does not directly quantify proton leak, respiration, mitochondrial abundance, or coupling efficiency, this result is not interpreted as direct evidence that DHM functions as a mitochondrial uncoupler.

To provide an additional assessment of mitochondrial content, the relative mtDNA copy number was quantified by qPCR in a separate cohort of Day-1 adult worms treated with 100 μM DHM for 48 h. DHM treatment did not significantly alter the mtDNA copy number relative to vehicle (1.048 ± 0.054-fold; one-sample *t*-test against a theoretical value of 1.0, *t*(4) = 0.90, *p* = 0.421, *n* = 5 independent biological replicates). Because this assay used a different developmental stage, DHM concentration range, and exposure duration than the TMRE experiment, and because the mtDNA copy number is an indirect proxy for mitochondrial mass, this non-significant result does not rule out a contribution of altered mitochondrial abundance or dye handling to the reduced TMRE signal. We therefore interpret the TMRE findings as consistent with, rather than definitive evidence of, an altered mitochondrial energetic state, and we identify a matched-protocol assessment of mitochondrial content as a priority for future work.

### 3.7. DHM Increases Mitochondrial Delivery to Acidic Compartments in ALM Neurons

**Figure 7:**
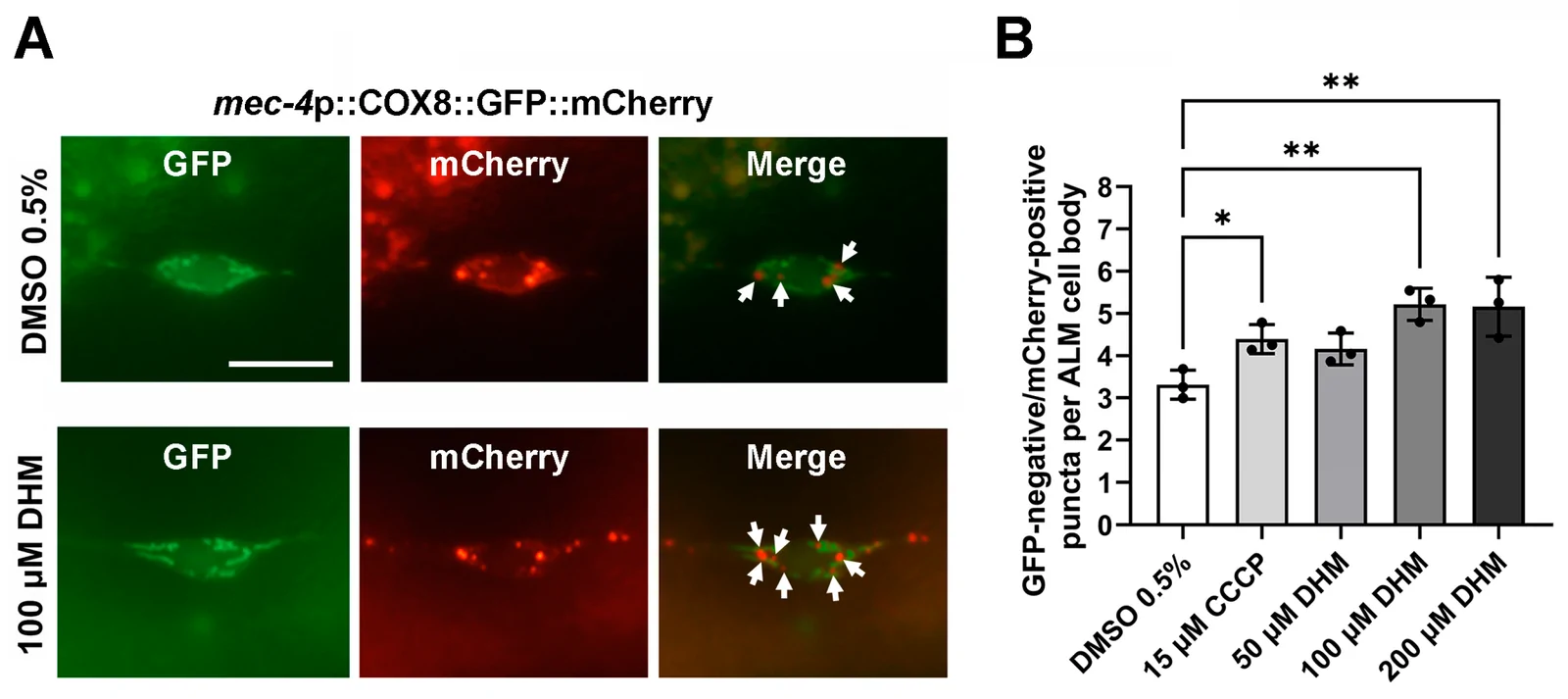
Dihydromyricetin increases GFP-negative/mCherry-positive mitochondrial puncta in ALM mechanosensory neurons of *C. elegans*. (**A**) Representative dual-channel fluorescence micrographs of day-3 adult *mec-4*p::COX8::GFP::mCherry worms treated with vehicle (0.5% DMSO) or 100 μM DHM, showing the ALM cell body (GFP, mCherry, and merged channels). Arrows indicate GFP-negative/mCherry-positive puncta. Scale bar = 10 μm. (**B**) Quantification of GFP-negative/mCherry-positive puncta per ALM cell body in vehicle, 15 μM CCCP, 50 μM DHM, 100 μM DHM, and 200 μM DHM groups. Mean ± SEM per ALM cell body: vehicle, 3.31 ± 0.20; CCCP, 4.39 ± 0.20; 50 μM DHM, 4.16 ± 0.22; 100 μM DHM, 5.22 ± 0.22; 200 μM DHM, 5.16 ± 0.40. Dunnett’s multiple-comparisons test versus vehicle used the biological replicate (*n* = 3) as the unit of analysis. Fifty ALM neurons were scored per treatment in each replicate (150 neurons per treatment across three experiments). Circles indicate each replicate mean. Only ALM cell bodies were scored; PLM neurons were not quantified. The puncta-based readout is interpreted as mitophagy-associated mitochondrial delivery rather than a direct quantitative measure of complete mitophagic flux. * *p* < 0.05, ** *p* < 0.01.

Mitochondrial delivery to acidic degradative compartments was evaluated in ALM mechanosensory neurons using the *mec-4*p::COX8::GFP::mCherry tandem reporter (Figure 7). GFP-negative/mCherry-positive puncta were quantified as mitochondrial structures delivered to acidic degradative compartments. Mean puncta counts per ALM cell body were 3.31 ± 0.20 in vehicle-treated worms, 4.39 ± 0.20 with 15 μM CCCP, 4.16 ± 0.22 with 50 μM DHM, 5.22 ± 0.22 with 100 μM DHM, and 5.16 ± 0.40 with 200 μM DHM (one-way ANOVA, *F*(4,10) = 9.269, *p* = 0.0021, *R*^2^ = 0.788). Using the biological replicate (*n* = 3) as the statistical unit, Dunnett’s multiple-comparisons test versus vehicle showed that CCCP increased puncta abundance by approximately 33% (*p* = 0.045), while 100 and 200 μM DHM increased puncta abundance by approximately 58% (*p* = 0.0013) and 56% (*p* = 0.0017), respectively. The 50 μM group showed a consistent upward trend (~26% increase) that did not reach significance (*p* = 0.126). These data are consistent with increased mitophagy-associated mitochondrial delivery to acidic compartments, but static puncta abundance does not directly quantify complete mitophagic flux. Data points in panel B represent the mean value of each independent biological replicate (*n* = 3), the unit used for statistical analysis.

### 3.8. DHM Neuroprotection Is Retained in ucp-4 Mutants but Is Not Statistically Detected in pink-1; pdr-1 Mutants

These two mutant backgrounds were selected to probe distinct, partially overlapping mitochondrial quality-control mechanisms potentially relevant to DHM action: *ucp-4* encodes the *C. elegans* uncoupling-protein homolog implicated in mitochondrial proton leak and stress-related neuronal phenotypes, making it a candidate mediator if DHM acted primarily through mild uncoupling, whereas *pink-1*; *pdr-1* encode the core PINK1/Parkin mitophagy machinery, allowing us to ask whether DHM neuroprotection instead depends on canonical damage-induced mitophagy. To examine whether mitochondrial regulatory backgrounds modified the neuroprotective phenotype, age-associated neuronal degeneration was analyzed in zdIs5; *ucp-4*(ok195) and zdIs5; *pink-1*(tm1779); *pdr-1*(gk448) backgrounds (Figure 2E–H). Vehicle-treated *ucp-4*(ok195) animals exhibited greater age-associated ALM and PLM degeneration than wild-type animals, yet DHM suppressed neuronal defects in this background, indicating that a statistically detectable DHM response can occur in the absence of UCP-4. In contrast, DHM did not significantly reduce ALM or PLM defects in *pink-1*; *pdr-1* double mutants at the examined ages. Because the available analysis was performed separately within each genotype, these results do not establish a significant genotype-by-treatment interaction. The lack of a statistically detectable response in the double-mutant background is therefore treated as a hypothesis-generating pattern compatible with, but not proof of, involvement of PINK-1/PDR-1-associated mitochondrial quality-control processes.

### 3.9. DHM Reduces Intestinal Lipid Accumulation

**Figure 8:**
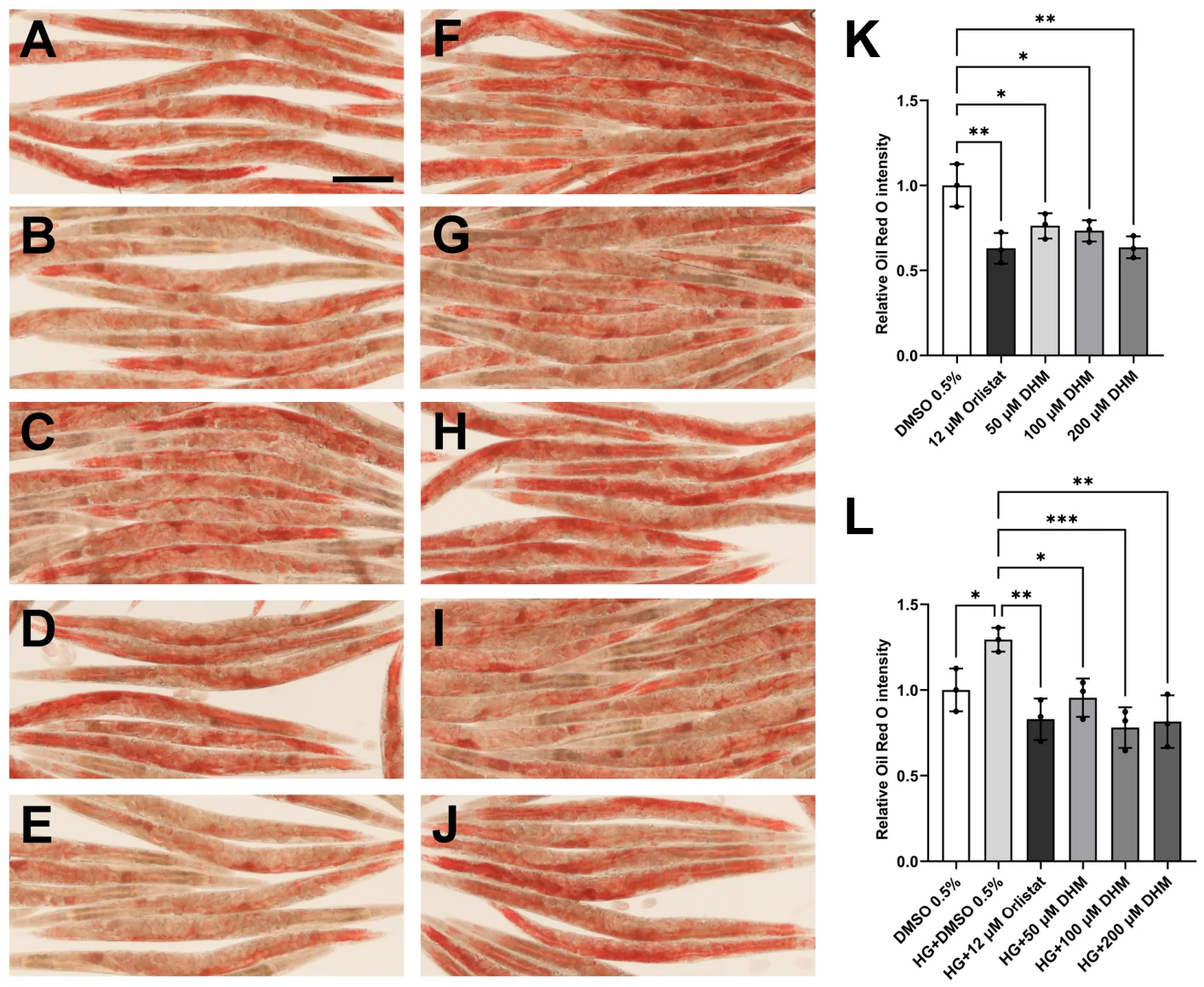
Dihydromyricetin reduces intestinal fat accumulation in *C. elegans*. (**A**–**E**) Representative Oil Red O-stained animals under basal conditions: vehicle (0.5% DMSO), 12 μM Orlistat, and DHM at 50, 100, and 200 μM. (**F**–**J**) Representative images under high-glucose conditions: 2% glucose + vehicle (HG), HG + 12 μM Orlistat, and HG co-treated with DHM at 50, 100, and 200 μM. Scale bar = 100 μm. (**K**) Quantification of relative Oil Red O intensity under basal conditions. (**L**) Quantification under high-glucose conditions. Data are presented as mean ± SEM from three independent experiments, with at least 70 worms per group. Circles indicate each replicate mean. Statistical analysis was performed using one-way ANOVA followed by Dunnett’s multiple comparisons versus DMSO (**K**) or HG + DMSO (**L**). * *p* < 0.05, ** *p* < 0.01, *** *p* < 0.001.

Having identified mitochondrial quality-control-associated changes, we next examined whether DHM also remodels metabolic homeostasis. Oil Red O staining was used to evaluate intestinal lipid storage (*N* > 70 per group; Figure 8). Under basal conditions, DHM reduced lipid accumulation by approximately 23.8% at 50 μM (*p* = 0.0229), 26.7% at 100 μM (*p* = 0.0117), and 36.4% at 200 μM (*p* = 0.0014), while the Orlistat positive control reduced staining by approximately 37.0% (*p* = 0.0013). High-glucose exposure increased lipid accumulation by approximately 29.4% above the vehicle baseline (*p* = 0.0407). DHM co-treatment significantly attenuated high-glucose-induced fat storage, reducing Oil Red O intensity by approximately 26.2% at 50 μM (*p* = 0.0180), 39.7% at 100 μM (*p* = 0.0008), and 36.9% at 200 μM (*p* = 0.0015) versus HG + DMSO. Thus, DHM remodels lipid storage under both basal and nutrient-excess conditions.

### 3.10. DHM Selectively Remodels Mitochondrial Stress and Lipid-Metabolism Gene Expression

Targeted RT-qPCR profiling was performed after 48 h treatment with 100 μM DHM across a panel of 22 genes related to IIS/FOXO signaling, mitochondrial stress adaptation, mitophagy, antioxidant defense, and lipid metabolism (Table S9). After Šidák correction for multiple comparisons, most genes were unchanged. The strongest transcriptional responses occurred in lipid metabolism: *fat-5* (0.58-fold; adjusted *p* = 0.0012), *fat-6* (0.52-fold; adjusted *p* = 0.0001), and *fat-7* (0.45-fold; adjusted *p* < 0.0001) were significantly downregulated. Among mitochondrial stress-adaptation genes, *atfs-1* was modestly increased (1.32-fold; adjusted *p* = 0.0389), whereas *hsp-6* and *hsp-60* were not significantly induced. No other tested IIS/FOXO, mitophagy, or canonical antioxidant-defense gene reached significance after correction. The selective suppression of Δ9 fatty-acid desaturase genes is directionally consistent with the reduced Oil Red O staining, whereas the isolated *atfs-1* response suggests a restrained mitochondrial stress-adaptive signal rather than broad activation of the canonical UPR^mt^ program.

### 3.11. DHM Alters DAF-16 Localization Without Canonical Reporter Activation, and Lifespan Extension Persists in daf-16 Mutants

**Figure 9:**
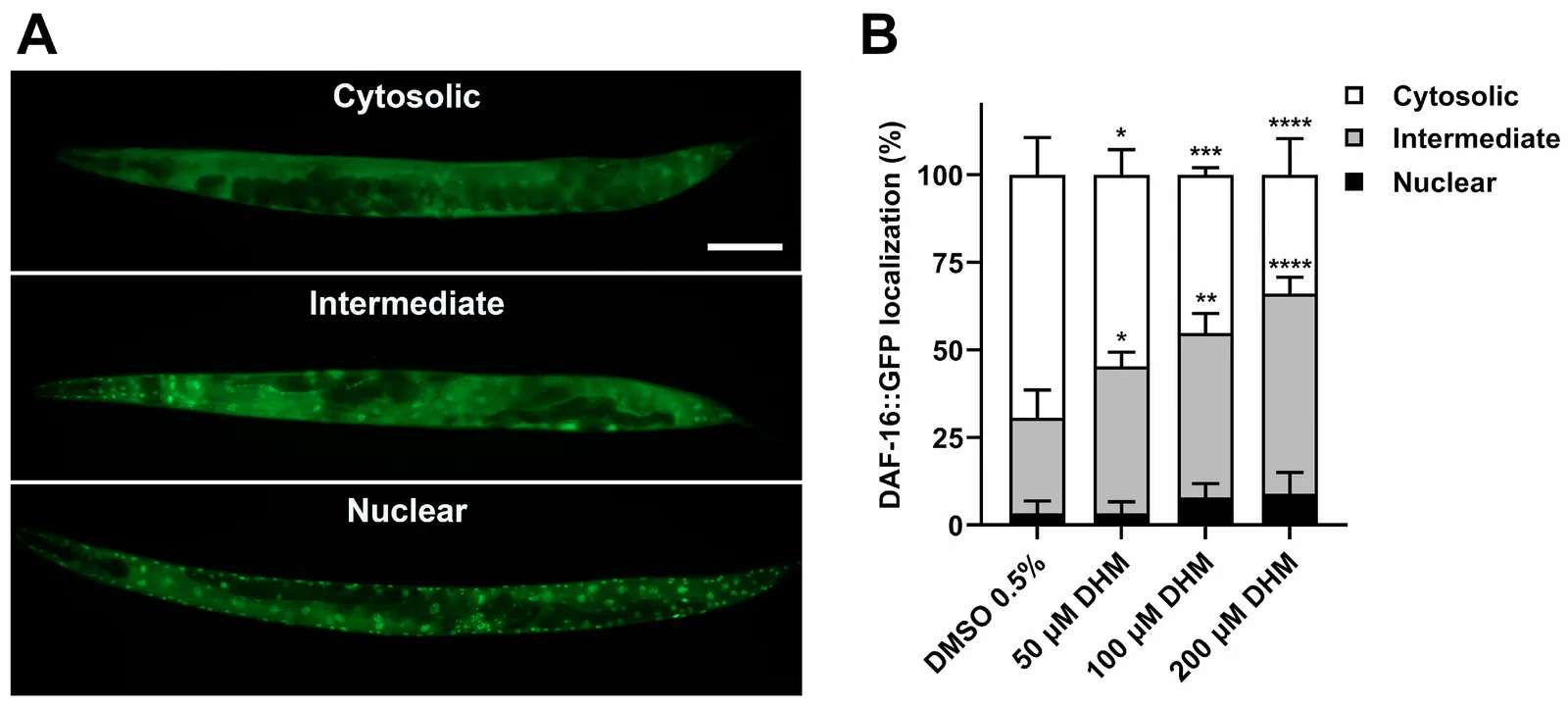
Dihydromyricetin alters DAF-16::GFP subcellular localization in *C. elegans*. (**A**) Representative fluorescence images of day-1 adult TJ356 DAF-16::GFP worms treated with vehicle or DHM (50, 100, or 200 μM) for 24 h, illustrating cytosolic, intermediate, and nuclear localization patterns. Scale bar = 100 μm. (**B**) Quantification of DAF-16::GFP localization. DHM shifted animals away from the cytosolic state and toward the intermediate category, whereas the fully nuclear fraction did not increase significantly. Data represent mean ± SEM from three independent biological replicates (replicate sizes ranged from 30 to 42 animals; *N* = 418 animals total). The two-way ANOVA/Dunnett category-wise analysis is presented as exploratory because the three localization percentages are compositional. * *p* < 0.05, ** *p* < 0.01, *** *p* < 0.001, **** *p* < 0.0001.

DAF-16::GFP localization was evaluated after 24 h DHM treatment (Figure 9). Vehicle-treated animals were predominantly cytosolic (69.3 ± 6.2%), with 27.3 ± 4.6% showing intermediate localization and 3.3 ± 2.1% showing fully nuclear localization. DHM shifted the distribution away from the cytosolic state at all three concentrations tested (adjusted *p* = 0.0250 at 50 μM, *p* = 0.0003 at 100 μM, *p* < 0.0001 at 200 μM), decreasing the cytosolic fraction to 54.7 ± 4.2% at 50 μM, 45.1 ± 1.2% at 100 μM, and 33.9 ± 6.0% at 200 μM, while correspondingly increasing the intermediate fraction to 42.0 ± 2.3% at 50 μM (adjusted *p* = 0.0250), 46.9 ± 3.2% at 100 μM (adjusted *p* = 0.0027), and 57.1 ± 2.7% at 200 μM (adjusted *p* < 0.0001). The fully nuclear fraction increased only modestly at each concentration and did not reach significance (all adjusted *p* > 0.5). In contrast to DAF-16::GFP, SKN-1::GFP localization after 14 h treatment (Figure S2; Table S11) was not significantly altered by DHM at any concentration: the nuclear, intermediate, and cytosolic fractions were all comparable with vehicle in the exploratory category-wise analysis (all Dunnett-adjusted *p* > 0.05). Because localization categories are compositional, these category-wise *p* values are interpreted descriptively and do not substitute for a formal multinomial or ordinal model. To examine the ordered categories, these data were additionally analyzed using an exploratory ordinal logistic regression (proportional-odds model), treating localization category as the ordinal outcome and DHM concentration as a continuous predictor. This pooled analysis suggested a concentration-dependent shift toward intermediate/nuclear localization (odds ratio = 2.01 per 100 μM increase in DHM concentration; *z* = 5.30, *p* < 0.0001, *N* = 418 animals from three independent biological replicates per condition: vehicle, 35/42/38; 50 μM DHM, 30/30/33; 100 μM DHM, 35/35/32; 200 μM DHM, 35/33/40), with a concordant linear-by-linear trend test on the same pooled data (χ^2^(1) = 27.35, *p* < 0.0001). Because this model treated individual animals rather than replicate means as the unit of analysis and did not include an explicit clustering or random-effects term for biological replicate, it is presented as a supporting, exploratory analysis rather than an independent confirmation of the replicate-level ANOVA reported above. Despite the DAF-16 localization shift, canonical downstream antioxidant reporter outputs were not significantly induced. *sod-3*p::GFP fluorescence remained at approximately 115–118% of control and *gst-4*p::GFP at approximately 84–92% of control, with no significant differences versus vehicle (Table S10). Consistent with these reporter results, *sod-3* and *gst-4* transcripts were not significantly altered in the RT-qPCR panel. To test functional requirement of DAF-16 for longevity, lifespan assays were performed in *daf-16*(mu86) mutants (Figure S1). DHM increased mean lifespan from 14.99 ± 0.22 days in vehicle-treated mutants to 15.82 ± 0.18, 16.42 ± 0.22, and 16.15 ± 0.21 days at 50, 100, and 200 μM, respectively, corresponding to increases of approximately 5.5–9.5% (all log-rank *p* < 0.0001). To formally test whether DAF-16 modifies the magnitude of this response, a Cox proportional hazards model was fit to the combined wild-type and *daf-16*(mu86) survival data, including genotype, DHM concentration (continuous), and a genotype × concentration interaction term. Both genotype (HR = 4.02, *p* < 0.005) and DHM concentration (HR = 0.79 per 100 μM, *p* < 0.005) were significant predictors of survival, consistent with the shorter baseline lifespan of *daf-16* mutants and the dose-dependent lifespan extension conferred by DHM. The genotype × concentration interaction term was not significant (*p* = 0.56); within the limits of this unclustered model, we did not detect evidence that the DHM dose–response relationship differed between wild-type and *daf-16*(mu86) backgrounds. Thus, DHM alters DAF-16 localization in a concentration-dependent manner without a detectable redistribution of SKN-1 or broad activation of the canonical SOD-3/GST-4 antioxidant reporter outputs, and a genotype × treatment interaction analysis did not detect evidence that the pro-longevity response is modified by *daf-16* status.

## 4. Discussion

No single mechanism accounted for all of DHM’s effects observed here. Lifespan was extended, age-related mechanosensory neurodegeneration was reduced, locomotor capacity held up into late adulthood, survival improved following acute juglone exposure, and intestinal lipid storage decreased. Underlying these organism-level outcomes were a modest shift in mitochondrial energetic state, increased mitochondrial delivery to acidic compartments, and selective changes in stress-response and lipid-metabolism transcripts—together suggesting coordinated redox and mitochondrial quality-control activity rather than a single dominant antioxidant pathway.

Because much of the current assay panel mirrors our earlier morin study [23], a direct (if qualitative) comparison is possible. Morin produced a pronounced TMRE decline linked to DAF-16-dependent mitochondrial stress adaptation. DHM’s profile differed: a smaller energetic shift, no detectable activation of the SOD-3 or GST-4 reporters, and lifespan extension that persisted even in *daf-16*(mu86) mutants. What distinguished DHM instead was strong cell-free radical-scavenging activity, increased neuron-specific mitochondrial trafficking to acidic compartments, and coordinated suppression of *fat-5*, *fat-6*, and *fat-7*—two flavonoids producing two distinct biological signatures.

The case for DHM’s antioxidant activity rests on two lines of evidence. In cell-free assays, DHM exhibited substantial DPPH and ABTS radical-scavenging capacity; in vivo, it improved survival following acute juglone exposure. Comparable links between direct redox activity and stress resistance have been reported for other phenolic antioxidants in *C. elegans* [40], and DHM’s radical-scavenging capacity has independently been confirmed in other cell-free oxidative systems [41]. Neither *sod-3* nor *gst-4* was significantly induced at the transcript or reporter level, making broad activation of these two canonical DAF-16/FOXO- and SKN-1/Nrf2-regulated outputs an unlikely explanation for the observed stress resistance. Direct chemical scavenging and altered mitochondrial redox balance remain plausible contributors, though this study cannot determine their relative contributions. It should also be noted that the cell-free DPPH/ABTS assays and the increased survival following acute juglone challenge demonstrate antioxidant capacity and stress resistance, respectively, but neither directly measures the in vivo redox or oxidative status of DHM-treated animals under basal aging conditions; direct in vivo markers of oxidative stress (e.g., protein carbonylation, lipid peroxidation, or ROS imaging) were not assessed in this study, and this is noted as a limitation of the present redox characterization.

TMRE fluorescence declined modestly in a concentration-dependent manner. Moderate shifts in mitochondrial energetics of this kind can trigger mitohormetic stress-adaptive responses [42], and reduced membrane potential has been observed across several *C. elegans* longevity paradigms [43]. As a partial, indirect check on mitochondrial content, the mtDNA copy number was also measured in a separate cohort and did not differ significantly from vehicle (*p* = 0.421); however, this assay used a different developmental stage, DHM concentration, and exposure duration than the TMRE experiment, so it cannot rule out a contribution of altered mitochondrial abundance to the TMRE signal. A further limitation is intrinsic to the TMRE assay itself: TMRE signal reflects not only membrane potential but also respiration, mitochondrial abundance, substrate handling, and ion balance, so it cannot alone isolate increased proton conductance from these other contributing factors [44,45]. The TMRE findings are consistent with altered mitochondrial membrane potential, but do not establish the cause of the reduced fluorescence or prove that DHM is a mitochondrial uncoupler.

A second neuron-level readout came from the tandem mitochondrial GFP::mCherry reporter, where DHM increased GFP-negative/mCherry-positive puncta in ALM cell bodies—consistent with greater mitochondrial delivery to acidic degradative compartments. Because mitophagy and mitochondrial biogenesis are co-regulated during *C. elegans* aging [46], this pattern could reflect enhanced clearance of dysfunctional mitochondria. Still, a static puncta count is a coarse measure: it is also influenced by mitochondrial abundance, lysosomal residence time, reporter expression level, and clearance kinetics. Direct assessment of complete mitophagic flux—for example, via mt-Keima-based ratiometric imaging or biochemical measurement of mitochondrial protein turnover—was not performed in this study and represents a priority for future work. What the data show is increased delivery to acidic compartments, not a quantified measure of complete mitophagic flux.

Testing genetic backgrounds added nuance without fully resolving the underlying pathway. In *ucp-4*(ok195) animals, DHM’s neuroprotective effect remained statistically detectable, consistent with a response that does not strictly require functional UCP-4—notable given that UCP-family proteins shape mitochondrial proton conductance and redox responses [47], and that UCP-4 itself has documented links to age-related neuronal phenotypes in *C. elegans* [48]. By contrast, neuroprotection was not statistically detected in *pink-1*(tm1779); *pdr-1*(gk448) double mutants. Because the two genotypes were analyzed separately without a formal genotype-by-treatment interaction test, this contrast alone cannot establish pathway dependency; it is compatible with a contributing role for PINK-1/PDR-1-mediated quality control. Testing the tandem mitochondrial reporter directly in the *pink-1*; *pdr-1* background—which was not performed in this study—would test whether PINK-1/PDR-1 is required for the DHM-associated increase in reporter puncta, rather than being inferred solely from the neuroprotection phenotype, and represents a clear priority for follow-up work. Additionally, the lifespan assay was performed in the presence of 50 μM FUdR to prevent progeny production; FUdR itself is known to affect nucleotide metabolism and stress-response pathways in *C. elegans*, so a contribution of FUdR to the lifespan phenotype cannot be fully excluded. The oxidative-stress and touch-response assays did not include FUdR, so this potential confound does not apply to those endpoints.

These molecular observations aligned with outcomes that matter functionally for the animal. DHM did more than extend lifespan—it also reduced structural abnormalities in aging mechanosensory neurons and preserved locomotor and anterior touch-response performance at adult days 7–9, providing direct functional support alongside the morphological findings. Functional validation in this study was limited to anterior touch responsiveness, which reflects both ALM and AVM function, following the anterior-touch design used in our previous mitochondrial uncoupling studies [30] and consistent with recent work assessing age-related mechanosensory decline via anterior touch alone [29]; PLM-mediated posterior touch responsiveness was not assessed, and future work should extend this functional assay to confirm whether the morphological preservation observed in PLM neurons corresponds to preserved function. Since branching, waviness, blebbing, and related structural changes mark progressive failure of neuronal maintenance in aging *C. elegans* [25,26], the observed neuronal, behavioral, stress-resistance, and mitochondrial readouts changed together, without establishing which changes mediated the others. The mitochondrial quality-control readouts were not all assessed at the same time point: TMRE staining began at L4 (6 h pretreatment followed by 4 h with dye and approximately 2 h washout); the tandem GFP::mCherry reporter was imaged on adult day 3 after three days of treatment initiated at L4. The mtDNA copy number and RT-qPCR results were measured after 48 h of treatment initiated on adult day 1. The neuroprotective and locomotor/touch-response phenotypes became most apparent at later adult ages (day 7 onward). The measurements were made in separate cohorts and at different ages; the temporal relationship within individual animals and any causal contribution of the mitochondrial changes to later neuroprotection remain untested.

DAF-16 and SKN-1 responded differently to DHM treatment. DAF-16::GFP shifted away from a predominantly cytosolic pattern toward intermediate localization, although the fully nuclear fraction did not increase significantly. A pooled ordinal logistic regression suggested a concentration-dependent shift away from the cytosolic state (odds ratio = 2.01 per 100 μM; model *p* < 0.0001). Because biological replicate clustering and the proportional-odds assumption were not addressed, its *p* value is exploratory and may be too small. SKN-1::GFP, by contrast, showed no statistically detectable change in nuclear, intermediate, or cytosolic distribution at any tested concentration, despite the fact that SKN-1 localization and target selection are known to shift with stress and signaling context [49,50]. The *sod-3*p::GFP and *gst-4*p::GFP reporters likewise remained unchanged, and neither *sod-3* nor *gst-4* transcript levels rose significantly. None of this supports a canonical SKN-1-to-GST-4 response under the conditions tested here, and because lifespan extension persisted in *daf-16*(mu86) mutants—with no genotype × dose interaction detected in the specified Cox model (*p* = 0.56; equivalence was not established)—a simple DAF-16-to-SOD-3 or SKN-1-to-GST-4 transcriptional model cannot fully account for the pro-longevity effect either—other pathways may also contribute. (The pooled trend test uses the same animals and does not provide independent validation of the ordinal model.)

Lipid handling shifted in parallel with these changes. Intestinal lipid accumulation decreased under both basal and high-glucose conditions, matched by coordinated downregulation of *fat-5*, *fat-6*, and *fat-7*—the Δ9 fatty-acid desaturases positioned within NHR-49-dependent metabolic regulation [51] and recognized as major determinants of adiposity and fatty-acid composition in *C. elegans* [52,53]. Excess intestinal lipid storage has been linked to metabolic dysfunction and reduced lifespan in *C. elegans*, so the observed reduction in lipid accumulation may reflect improved metabolic homeostasis that is broadly consistent with the healthspan-related phenotypes reported here, although this study did not test whether the lipid changes are causally required for the lifespan or neuroprotective effects of DHM, and they are presented as a correlative component of the overall metabolic remodeling. Both the Oil Red O staining and transcriptional data point in the same direction. DHM also produced a modest increase in *atfs-1* without significantly inducing *hsp-6* or *hsp-60*; since ATFS-1 signaling depends on mitochondrial import efficiency and helps coordinate stress adaptation [54,55], this pattern suggests a restrained stress-adaptive response rather than full canonical UPR^mt^ activation. Whether the lipid and mitochondrial changes are mechanistically connected remains an open question, since fatty-acid composition, β-oxidation flux, and mitochondrial respiration were not directly measured in this study.

Several limitations are worth stating plainly. The TMRE decrease alone does not establish mitochondrial uncoupling, which would require direct measurement of oxygen consumption, ATP production, proton leak, and mitochondrial abundance; the mtDNA copy number was measured as a partial check for this and did not differ significantly between groups. The tandem reporter captures puncta-based delivery to acidic compartments rather than complete mitophagic flux; direct assessment of flux—for example, via mt-Keima-based ratiometric imaging or biochemical measurement of mitochondrial protein turnover—was not performed in this study and represents a priority for future work. Neither mitochondrial membrane potential nor oxidative-burden/quality-control markers were directly measured in the *ucp-4* or *pink-1*; *pdr-1* backgrounds, and no formal cross-genotype interaction test was conducted for the neurodegeneration phenotype in these mutants; testing the tandem mitochondrial reporter directly in the *pink-1*; *pdr-1* background would test whether PINK-1/PDR-1 is required for the DHM-associated reporter response. Because ALM and PLM represent only a limited subset of mechanosensory neurons, their morphological preservation should not be generalized to neuroprotection across the nervous system. Functional validation was limited to anterior touch responsiveness, which reflects both ALM and AVM function at adult days 3–9; PLM-mediated posterior touch responsiveness was not assessed, and measurements were not extended to day 15 because preliminary observations showed near-complete loss of touch responsiveness across all groups at that age (a floor effect consistent with the sharp decline in *C. elegans* locomotory function after day 10 of adulthood [27,28]). The DAF-16/SKN-1 localization scoring reflects a three-category compositional outcome; this was supplemented with a pooled ordinal logistic regression and a trend test on the same animals; neither analysis adjusted for biological replicate clustering, and the proportional-odds assumption was not formally checked, so the resulting *p* values remain exploratory. Similarly, while the *daf-16*(mu86) and wild-type lifespan analyses were initially performed separately, a Cox proportional hazards model with a genotype × dose interaction term did not detect a difference in the DHM dose–response relationship between genotypes; equivalence was not established. Finally, all findings derive from *C. elegans*, and whether they extend to mammalian systems remains untested.

Taken together, the results are consistent with a model in which DHM’s antioxidant capacity and stress-resistance phenotype are accompanied by a broader mitochondrial quality-control response—lower TMRE fluorescence accompanied by increased mitochondrial reporter puncta in acidic compartments, functionally supported by preserved locomotor and anterior touch-response performance. The persistence of neuroprotection in *ucp-4* mutants argues against a strict dependence on UCP-4, while its absence in *pink-1*; *pdr-1* mutants remains hypothesis-generating rather than conclusive pending direct interaction testing. Alongside this, DHM’s suppression of Δ9 desaturase expression and intestinal lipid storage points to systemic metabolic remodeling rather than an isolated effect. Redox homeostasis and mitochondrial quality control anchor this overall framework, with individual genetic pathways and mild uncoupling remaining plausible contributing features rather than fully established, singular mechanisms.

## 5. Conclusions

DHM is associated with healthspan-related phenotypes in *C. elegans*, including extended lifespan alongside preserved neuronal integrity, locomotor and anterior touch-response function, oxidative-stress resistance, and lipid balance. Its antioxidant phenotype is supported by direct radical-scavenging activity and improved survival under juglone exposure, occurring despite the absence of significant induction of the canonical SOD-3 or GST-4 reporter outputs.

DHM reduced TMRE fluorescence and increased mitochondrial delivery to acidic degradative compartments within mechanosensory neurons, a pattern consistent with increased mitophagy-associated delivery but insufficient to demonstrate turnover or complete flux. Separately timed mtDNA analysis did not detect a significant copy-number difference, so these data do not isolate a change in membrane potential from a change in mitochondrial mass. Combined with the selective *atfs-1* response, these findings are consistent with mitochondrial quality-control changes accompanying neuroprotection, without establishing mediation. This neuroprotective effect remained detectable in *ucp-4* mutants but was not statistically detected in *pink-1*; *pdr-1* mutants; absent a formal genotype-by-treatment interaction test, the latter finding is interpreted as supportive, hypothesis-generating evidence rather than definitive proof of pathway dependency. In parallel, suppression of *fat-5*, *fat-6*, and *fat-7* accompanied the reduction in intestinal lipid storage. Overall, redox-related, mitochondrial, and lipid-related readouts changed alongside the observed healthspan-related phenotypes; their causal relationships remain unresolved.

## Figures and Tables

**Table 1:** Lifespan statistics of *C. elegans* under DHM treatment.

Group	*N*	Mean Lifespan (Days)	Median Lifespan (Days)	Max Lifespan (Days)	*p*-Value vs. CONTROL	Change (%)
Control (0.5% DMSO)	441	19.96 ± 0.31	20.5	33	—	—
50 μM DHM	446	21.78 ± 0.32	22	35	<0.0001	+9.1
100 μM DHM	453	22.45 ± 0.33	23	37	<0.0001	+12.5
200 μM DHM	442	22.96 ± 0.34	24	36	<0.0001	+15.0

Data represent pooled results from three independent assays (mean ± SEM). Statistical significance was determined by log-rank (Mantel–Cox) tests against the vehicle control. DHM, dihydromyricetin; DMSO, dimethyl sulfoxide.

## Data Availability

The original contributions presented in this study are included in the article/Supplementary Materials. Further inquiries can be directed to the corresponding author.

## Supplementary Materials

The following supporting information can be downloaded at: https://www.mdpi.com/article/10.3390/antiox15101251/s1, Table S1: Kaplan–Meier survival data and log-rank statistics for wild-type (N2) animals (*daf-16*(mu86) survival data are reported in the main text, Section 3.11, and Figure S1); Table S2: Neurodegeneration statistics; Table S3: Locomotion statistics; Table S4: Anterior touch-response statistics; Table S5: ALM GFP-negative/mCherry-positive puncta quantification statistics; Table S6: Oil Red O quantification statistics; Table S7: TMRE quantification statistics; Table S8: RT-qPCR primer sequences; Table S9: RT-qPCR gene-expression fold changes and statistics; Table S10: *sod-3*p::GFP and *gst-4*p::GFP quantification statistics; Table S11: SKN-1::GFP subcellular localization statistics; Table S12: mitochondrial DNA copy number statistics; Figure S1: Kaplan–Meier survival curves of *daf-16*(mu86) animals treated with DHM; Figure S2: SKN-1::GFP localization following DHM treatment.
