# Supplementary material for: Dihydromyricetin Is Associated with Healthspan-Related Phenotypes and Neuroprotection in Caenorhabditis elegans, Alongside Redox and Mitochondrial Quality-Control Changes
Source: Antioxidants (Basel). 2026 Sep 30;15(10):1251. doi: 10.3390/antiox15101251 (PMC13700736; doi:10.3390/antiox15101251)
Supplement: Supplementary file 1 [file antioxidants-15-01251-s001.zip › antioxidants-4570268-supplementary.pdf]

# Supplementary Materials

## Dihydromyricetin Is Associated with Healthspan-Related Phenotypes and Neuroprotection in *Caenorhabditis elegans*, Alongside Redox and Mitochondrial Quality-Control Changes

**Supplementary Table S1.** Kaplan–Meier survival analysis and log-rank statistics for wild-type (N2) worms (related to Figure 1; Table 1)

Kaplan–Meier survival curves of wild-type (N2) worms treated with vehicle (0.5% DMSO) or DHM (50, 100, or 200 µM) were compared using the log-rank (Mantel–Cox) test. Individual pairwise comparisons versus vehicle were not corrected for multiple comparisons. Data are pooled from three independent biological replicates.

**A. Log-rank (Mantel–Cox) test — overall comparison (all four groups)**

| Chi square (df) | P value | Significance |
|---|---|---|
| 82.35 (3) | <0.0001 | **** |

**B. Log-rank test — pairwise comparisons vs. vehicle (uncorrected)**

| Comparison | Median 1 vs. 2 (days) | Chi square (df=1) | P value | Sig. |
|---|---|---|---|---|
| DMSO 0.5% vs. 50 µM DHM | 20.5 vs. 22 | 26.38 | <0.0001 | **** |
| DMSO 0.5% vs. 100 µM DHM | 20.5 vs. 23 | 48.05 | <0.0001 | **** |
| DMSO 0.5% vs. 200 µM DHM | 20.5 vs. 24 | 70.19 | <0.0001 | **** |

N (Day 0): 441 (DMSO 0.5%), 446 (50 µM DHM), 453 (100 µM DHM), 442 (200 µM DHM). Deaths/events: 436, 441, 448, 437, respectively (5 censored subjects per group). Kaplan–Meier survival curves and log-rank statistics for *daf-16*(mu86) mutant assays are reported in the main text (Section 3.11) and Figure S1.

**Supplementary Table S2.** Statistical analysis of neurodegeneration assays (related to Figure 2)

ALM and PLM neuronal degeneration was analyzed in wild-type (zdIs5 [P*mec-4*::GFP]), zdIs5; *ucp-4*(ok195), and zdIs5; *pink-1*(tm1779); *pdr-1*(gk448) worms at adult days 5, 10, and 15. For each genetic background and neuron type, an ordinary two-way ANOVA (Age × Treatment) was performed, followed by Dunnett's multiple-comparisons test comparing each DHM concentration with vehicle within each age. n = 3 independent biological replicates per group. These within-genotype analyses do not constitute a formal genotype × treatment interaction test.

**A. Wild-type (zdIs5) — ALM, two-way ANOVA summary**

| Source of Variation | F (DFn, DFd) | P value | Significance |
|---|---|---|---|
| Interaction (Age × Treatment) | 3.545 (6,24) | 0.0118 | * |
| Age (main effect) | 12.01 (2,24) | 0.0002 | *** |
| Treatment (main effect) | 5.371 (3,24) | 0.0057 | ** |

**B. Wild-type (zdIs5) — ALM, Dunnett's post-hoc (vs. DMSO)**

| Age | Comparison (vs. DMSO) | Adjusted p-value | Significance |
|---|---|---|---|
| Day 5 | 50 µM DHM | 0.9469 | ns |
| Day 5 | 100 µM DHM | 0.3648 | ns |
| Day 5 | 200 µM DHM | 0.1446 | ns |
| Day 10 | 50 µM DHM | 0.1478 | ns |
| Day 10 | 100 µM DHM | 0.7392 | ns |
| Day 10 | 200 µM DHM | 0.9997 | ns |
| Day 15 | 50 µM DHM | 0.0636 | ns |
| Day 15 | 100 µM DHM | 0.0017 | ** |
| Day 15 | 200 µM DHM | 0.0029 | ** |

**C. Wild-type (zdIs5) — PLM, two-way ANOVA summary**

| Source of Variation | F (DFn, DFd) | P value | Significance |
|---|---|---|---|
| Interaction (Age × Treatment) | 2.942 (6,24) | 0.0269 | * |
| Age (main effect) | 88.23 (2,24) | <0.0001 | **** |

| Source of Variation | F (DFn, DFd) | P value | Significance |
|---|---|---|---|
| Treatment (main effect) | 7.611 (3,24) | 0.0010 | *** |

**D. Wild-type (zdIs5) — PLM, Dunnett's post-hoc (vs. DMSO)**

| Age | Comparison (vs. DMSO) | Adjusted p-value | Significance |
|---|---|---|---|
| Day 5 | 50 µM DHM | 0.8459 | ns |
| Day 5 | 100 µM DHM | 0.9306 | ns |
| Day 5 | 200 µM DHM | 0.9525 | ns |
| Day 10 | 50 µM DHM | 0.0009 | *** |
| Day 10 | 100 µM DHM | 0.0107 | * |
| Day 10 | 200 µM DHM | 0.2134 | ns |
| Day 15 | 50 µM DHM | 0.0282 | * |
| Day 15 | 100 µM DHM | 0.0027 | ** |
| Day 15 | 200 µM DHM | 0.0032 | ** |

**E. zdIs5; *ucp-4*(ok195) — ALM, two-way ANOVA summary**

| Source of Variation | F (DFn, DFd) | P value | Significance |
|---|---|---|---|
| Interaction (Age × Treatment) | 0.9350 (6,24) | 0.4883 | ns |
| Age (main effect) | 9.866 (2,24) | 0.0007 | *** |
| Treatment (main effect) | 15.01 (3,24) | <0.0001 | **** |

**F. zdIs5; *ucp-4*(ok195) — ALM, Dunnett's post-hoc (vs. DMSO)**

| Age | Comparison (vs. DMSO) | Adjusted p-value | Significance |
|---|---|---|---|
| Day 5 | 50 µM DHM | 0.0841 | ns |
| Day 5 | 100 µM DHM | 0.0032 | *** |
| Day 5 | 200 µM DHM | 0.1505 | ns |
| Day 10 | 50 µM DHM | 0.1308 | ns |
| Day 10 | 100 µM DHM | 0.0527 | ns |
| Day 10 | 200 µM DHM | 0.0067 | ** |
| Day 15 | 50 µM DHM | 0.0274 | * |
| Day 15 | 100 µM DHM | 0.0009 | *** |
| Day 15 | 200 µM DHM | 0.0006 | *** |

**G. zdIs5; *ucp-4*(ok195) — PLM, two-way ANOVA summary**

| Source of Variation | F (DFn, DFd) | P value | Significance |
|---|---|---|---|
| Interaction (Age × Treatment) | 0.7073 (6,24) | 0.6469 | ns |
| Age (main effect) | 97.17 (2,24) | <0.0001 | **** |
| Treatment (main effect) | 17.69 (3,24) | <0.0001 | **** |

**H. zdIs5; *ucp-4*(ok195) — PLM, Dunnett's post-hoc (vs. DMSO)**

| Age | Comparison (vs. DMSO) | Adjusted p-value | Significance |
|---|---|---|---|
| Day 5 | 50 µM DHM | 0.0355 | * |
| Day 5 | 100 µM DHM | 0.0191 | * |
| Day 5 | 200 µM DHM | 0.1330 | ns |
| Day 10 | 50 µM DHM | 0.0215 | * |
| Day 10 | 100 µM DHM | 0.0006 | *** |

| Age | Comparison (vs. DMSO) | Adjusted p-value | Significance |
|---|---|---|---|
| Day 10 | 200 µM DHM | 0.0174 | * |
| Day 15 | 50 µM DHM | 0.0101 | * |
| Day 15 | 100 µM DHM | 0.0005 | *** |
| Day 15 | 200 µM DHM | 0.0004 | *** |

**I. zdIs5; *pink-1*(tm1779); *pdr-1*(gk448) — ALM, two-way ANOVA summary**

| Source of Variation | F (DFn, DFd) | P value | Significance |
|---|---|---|---|
| Interaction (Age × Treatment) | 0.08794 (6,24) | 0.9970 | ns |
| Age (main effect) | 17.63 (2,24) | <0.0001 | **** |
| Treatment (main effect) | 0.6082 (3,24) | 0.6162 | ns |

**J. zdIs5; *pink-1*(tm1779); *pdr-1*(gk448) — ALM, Dunnett's post-hoc (vs. DMSO)**

| Age | Comparison (vs. DMSO) | Adjusted p-value | Significance |
|---|---|---|---|
| Day 5 | 50 µM DHM | 0.8253 | ns |
| Day 5 | 100 µM DHM | 0.9634 | ns |
| Day 5 | 200 µM DHM | 0.9884 | ns |
| Day 10 | 50 µM DHM | 0.9192 | ns |
| Day 10 | 100 µM DHM | 0.8700 | ns |
| Day 10 | 200 µM DHM | >0.9999 | ns |
| Day 15 | 50 µM DHM | 0.9393 | ns |
| Day 15 | 100 µM DHM | 0.8096 | ns |
| Day 15 | 200 µM DHM | 0.9192 | ns |

**K. zdIs5; *pink-1*(tm1779); *pdr-1*(gk448) — PLM, two-way ANOVA summary**

| Source of Variation | F (DFn, DFd) | P value | Significance |
|---|---|---|---|
| Interaction (Age × Treatment) | 0.1288 (6,24) | 0.9915 | ns |
| Age (main effect) | 80.86 (2,24) | <0.0001 | **** |
| Treatment (main effect) | 1.792 (3,24) | 0.1756 | ns |

**L. zdIs5; *pink-1*(tm1779); *pdr-1*(gk448) — PLM, Dunnett's post-hoc (vs. DMSO)**

| Age | Comparison (vs. DMSO) | Adjusted p-value | Significance |
|---|---|---|---|
| Day 5 | 50 µM DHM | 0.8562 | ns |
| Day 5 | 100 µM DHM | 0.9306 | ns |
| Day 5 | 200 µM DHM | 0.7605 | ns |
| Day 10 | 50 µM DHM | 0.3519 | ns |
| Day 10 | 100 µM DHM | 0.5992 | ns |
| Day 10 | 200 µM DHM | 0.6537 | ns |
| Day 15 | 50 µM DHM | 0.3111 | ns |
| Day 15 | 100 µM DHM | 0.7079 | ns |
| Day 15 | 200 µM DHM | 0.3111 | ns |

**Supplementary Table S3.** Statistical analysis of the locomotion assay (related to Figure 3)

Locomotor activity of wild-type (N2) worms was measured at adult days 3, 5, 7, and 9 using the WMicrotracker SMART system. An ordinary two-way ANOVA (Age × Treatment) was performed, followed by Dunnett's multiple-comparisons test comparing each DHM concentration with vehicle within each age. n = 3 independent biological replicates per group (≥30 worms per replicate).

**A. Two-way ANOVA summary**

| Source of Variation | F (DFn, DFd) | P value | Significance |
|---|---|---|---|
| Interaction (Age × Treatment) | 2.516 (9,32) | 0.0264 | * |
| Age (main effect) | 517.9 (3,32) | <0.0001 | **** |
| Treatment (main effect) | 3.016 (3,32) | 0.0442 | * |

**B. Dunnett's post-hoc (vs. DMSO)**

| Age | Comparison (vs. DMSO) | Adjusted p-value | Significance |
|---|---|---|---|
| Day 3 | 50 µM DHM | 0.8587 | ns |
| Day 3 | 100 µM DHM | 0.0815 | ns |
| Day 3 | 200 µM DHM | 0.2734 | ns |
| Day 5 | 50 µM DHM | 0.5841 | ns |
| Day 5 | 100 µM DHM | 0.3248 | ns |
| Day 5 | 200 µM DHM | 0.1016 | ns |
| Day 7 | 50 µM DHM | 0.9106 | ns |
| Day 7 | 100 µM DHM | 0.0404 | * |
| Day 7 | 200 µM DHM | 0.0146 | * |
| Day 9 | 50 µM DHM | 0.5841 | ns |
| Day 9 | 100 µM DHM | 0.0316 | * |
| Day 9 | 200 µM DHM | 0.1547 | ns |

### Supplementary Table S4. Statistical analysis of the anterior touch-response assay (related to Figure 3)

Anterior touch responsiveness (reflecting ALM and AVM function) of wild-type (N2) worms was assessed at adult days 3, 5, 7, and 9, using the same treatment groups as the locomotion assay. An ordinary two-way ANOVA (Age × Treatment) was performed, followed by Dunnett's multiple-comparisons test comparing each DHM concentration with vehicle within each age. n = 3 independent biological replicates per group per age.

**A. Two-way ANOVA summary**

| Source of Variation | F (DFn, DFd) | P value | Significance |
|---|---|---|---|
| Interaction (Age × Treatment) | 1.478 (9,32) | 0.1984 | ns |
| Age (main effect) | 39.31 (3,32) | <0.0001 | **** |
| Treatment (main effect) | 3.774 (3,32) | 0.0200 | * |

**B. Dunnett's post-hoc (vs. DMSO)**

| Age | Comparison (vs. DMSO) | Adjusted p-value | Significance |
|---|---|---|---|
| Day 3 | 50 µM DHM | 0.8616 | ns |
| Day 3 | 100 µM DHM | 0.9665 | ns |
| Day 3 | 200 µM DHM | 0.6985 | ns |
| Day 5 | 50 µM DHM | 0.9518 | ns |
| Day 5 | 100 µM DHM | 0.7680 | ns |
| Day 5 | 200 µM DHM | >0.9999 | ns |
| Day 7 | 50 µM DHM | 0.9665 | ns |
| Day 7 | 100 µM DHM | 0.0177 | * |
| Day 7 | 200 µM DHM | 0.0259 | * |
| Day 9 | 50 µM DHM | 0.9127 | ns |
| Day 9 | 100 µM DHM | 0.0477 | * |

| Age | Comparison (vs. DMSO) | Adjusted p-value | Significance |
|---|---|---|---|
| Day 9 | 200 μM DHM | 0.1055 | ns |

**Supplementary Table S5.** Statistical analysis of the tandem mitochondrial reporter assay (related to Figure 7)

GFP-negative/mCherry-positive puncta per ALM cell body were quantified in *mec-4*p::COX8::GFP::mCherry worms treated with vehicle (DMSO 0.5%), 15 μM CCCP, or DHM (50, 100, or 200 μM). An ordinary one-way ANOVA was performed, followed by Dunnett's multiple-comparisons test comparing each treatment with vehicle. n = 3 independent biological replicates per group; 50 ALM neurons were scored per treatment in each replicate (150 neurons per treatment across three experiments).

**A. One-way ANOVA summary**

| Source of Variation | F (DFn, DFd) | P value | Significance |
|---|---|---|---|
| Treatment (between groups) | 9.269 (4,10) | 0.0021 | ** |

**B. Dunnett's post-hoc (vs. DMSO 0.5%)**

| Comparison (vs. DMSO 0.5%) | Adjusted p-value | Significance |
|---|---|---|
| 15 μM CCCP | 0.0450 | * |
| 50 μM DHM | 0.1262 | ns |
| 100 μM DHM | 0.0013 | ** |
| 200 μM DHM | 0.0017 | ** |

**Supplementary Table S6.** Oil Red O quantification statistics (related to Figure 8)

Relative Oil Red O intensity was compared using one-way ANOVA followed by Dunnett's multiple-comparisons test versus vehicle (0.5% DMSO; basal conditions) or versus HG + vehicle (high-glucose conditions). Data represent the mean of three independent biological replicates (n = 3).

**A. Basal conditions — one-way ANOVA summary**

| F (DFn, DFd) | P value | Significance |
|---|---|---|
| F (4, 10) = 9.075 | 0.0023 | ** |

**B. Basal conditions — Dunnett's multiple comparisons vs. vehicle**

| Comparison | Mean diff. | 95% CI | Adjusted P value | Sig. |
|---|---|---|---|---|
| DMSO 0.5% vs. 12 μM Orlistat | 0.3697 | 0.166 to 0.574 | 0.0013 | ** |
| DMSO 0.5% vs. 50 μM DHM | 0.2377 | 0.034 to 0.442 | 0.0229 | * |
| DMSO 0.5% vs. 100 μM DHM | 0.2672 | 0.063 to 0.471 | 0.0117 | * |
| DMSO 0.5% vs. 200 μM DHM | 0.3643 | 0.160 to 0.568 | 0.0014 | ** |

**C. High-glucose conditions — one-way ANOVA summary**

| F (DFn, DFd) | P value | Significance |
|---|---|---|
| F (5, 12) = 7.682 | 0.0019 | ** |

**D. High-glucose conditions — Dunnett's multiple comparisons vs. HG + vehicle**

| Comparison | Mean diff. | 95% CI | Adjusted P value | Sig. |
|---|---|---|---|---|
| HG + DMSO vs. DMSO 0.5% (basal) | 0.2938 | 0.011 to 0.576 | 0.0407 | * |
| HG + DMSO vs. HG + 12 μM Orlistat | 0.4643 | 0.182 to 0.747 | 0.0019 | ** |
| HG + DMSO vs. HG + 50 μM DHM | 0.3385 | 0.056 to 0.621 | 0.0180 | * |
| HG + DMSO vs. HG + 100 μM DHM | 0.5135 | 0.231 to 0.796 | 0.0008 | *** |
| HG + DMSO vs. HG + 200 μM DHM | 0.4781 | 0.196 to 0.760 | 0.0015 | ** |

Means (normalized to basal DMSO 0.5% = 1.000): 12 μM Orlistat = 0.630, 50 μM DHM = 0.762, 100 μM DHM = 0.733, 200 μM DHM = 0.636; HG + DMSO = 1.294, HG + 12 μM Orlistat = 0.830, HG + 50 μM DHM = 0.955, HG + 100 μM DHM = 0.780, HG + 200 μM DHM = 0.816.

**Supplementary Table S7.** TMRE quantification statistics (related to Figure 6)

Whole-body TMRE fluorescence, normalized to the vehicle-treated mean, was compared using one-way ANOVA followed by Dunnett's multiple-comparisons test versus vehicle (0.5% DMSO). Data represent the mean of three independent biological replicates (n = 3).

**A. One-way ANOVA summary**

| F (DFn, DFd) | P value | Significance |
|---|---|---|
| F (4, 10) = 7.566 | 0.0045 | ** |

**B. Dunnett's multiple comparisons test vs. vehicle**

| Comparison | Mean diff. | 95% CI | Adjusted P value | Sig. |
|---|---|---|---|---|
| DMSO 0.5% vs. 50 μM CCCP | 0.2784 | 0.112 to 0.445 | 0.0023 | ** |
| DMSO 0.5% vs. 50 μM DHM | 0.0941 | −0.072 to 0.261 | 0.3473 | ns |
| DMSO 0.5% vs. 100 μM DHM | 0.2025 | 0.036 to 0.369 | 0.0180 | * |
| DMSO 0.5% vs. 200 μM DHM | 0.2263 | 0.060 to 0.393 | 0.0093 | ** |

Means (normalized to vehicle): DMSO 0.5% = 1.002, 50 μM CCCP = 0.724, 50 μM DHM = 0.908, 100 μM DHM = 0.800, 200 μM DHM = 0.776.

**Supplementary Table S8.** Primer sequences

| Category | Gene | Forward primer seq | Reverse primer seq |
|---|---|---|---|
| Control | *cdc-42* | 5'-CTGCTGGACAGGAAGATTACG-3' | 5'-CTCGGACATTCTCGAATGAAG-3' |
| Energy/IIS–FOXO & Longevity Regulators | *aak-2* | 5'-TGCTTCACCATATGCTCTGC-3' | 5'-GTGGATCATCTCCCAGCAAT-3' |
| | *daf-2* | 5'-GAGAGAATGATGTGCCAACG-3' | 5'-CACCACGATTTGTTAGGCAATA-3' |
| | *daf-16* | 5'-CCAGGAAGGAATCCACGGCGT-3' | 5'-TGGCTCCGCGGCGAGATTT-3' |
| | *skn-1* | 5'-GCCAGCGGACAACGGAAGC-3' | 5'-GGAACGCCGACACTGGAAGC-3' |
| | *sir-2.1* | 5'-TGGCTGACGATTCGATGGAT-3' | 5'-ATGAGCAGAAATCGCGACAC-3' |
| Mitochondrial stress adaptation | *atfs-1* | 5'-TCGGCGATCGATCAGCTAAC-3' | 5'-AGAATCAGTTCTTGGATTAGGGGA-3' |
| | *hsp-6* | 5'-GATTGGATAAGGACGCTGGAG-3' | 5'-GAGGAATGTATCTCCGTTGGTG-3' |
| | *hsp-60* | 5'-CACCGGAAACAGCAATACATC-3' | 5'-CGACTCCTGAAGCATCTTGG-3' |
| | *polg-1* | 5'-GGATGCCAAATTGCGGATTC-3' | 5'-CATTGAGACGAGCAGGAGATG-3' |
| | *fmo-2* | 5'-GTGGCAGTTGAGCAATCAAG-3' | 5'-TGAGGAAACAGTTTGTAGAGTGAG-3' |
| Mitophagy | *pink-1* | 5'-ACCTCTTCCAGCAAAACATCC-3' | 5'-GCAATTGACTCATACCAACGTG-3' |
| | *pdr-1* | 5'-TCACTGACACCTGCAACAC-3' | 5'-TGCACCACACGTAGAAGC-3' |
| Stress/Antioxidant defenses | *hsf-1* | 5'-AGAGATGCGTGCGATGCGAGA-3' | 5'-GGCGAGCATGTTGTTGACGCA-3' |
| | *hsp-16.2* | 5'-CTGCAGAATCTCTCCATCTGAGTC-3' | 5'-AGATTCGAAGCAACTGCACC-3' |
| | *gst-4* | 5'-TGATGCTCGTGCTCTTGCTG-3' | 5'-CATGGCTTCACTCACTTGGCT-3' |
| | *ctl-1* | 5'-AGCGTGTCGTTCATGCCAAGG-3' | 5'-CCGATTCTCCAGCGACCGTTG-3' |
| | *sod-3* | 5'-CATTGTTTCAGCGCGACTTCGG-3' | 5'-TCCCCAGCCAGAGCCTTGAAC-3' |
| Lipid metabolism | *acs-2* | 5'-AGATGGTGAAGGAGATGAGAATG-3' | 5'-TGGCGAGTTCTTGATGTGG-3' |
| | *nhr-49* | 5'-GATTCTCTGCTCTCCGAGTTC-3' | 5'-AAACACTTCCCACTGATCCG-3' |

| Category | Gene | Forward primer seq | Reverse primer seq |
|---|---|---|---|
| | *fat-5* | 5'-GATTTGTACGAGGATCCGGTG-3' | 5'-CGTGTAGAAGGCGATGAAGG-3' |
| | *fat-6* | 5'-ATTATCGGCCGGCAGGTATC-3' | 5'-TTTTCCTCGTTGAATATCACATCC-3' |
| | *fat-7* | 5'-GGAAGGAGACAGCATTCATTGCG-3' | 5'-GTCTTGTGGGAATGTGTGGTGG-3' |

**Supplementary Table S9.** RT-qPCR gene expression in DHM-treated *C. elegans* (22-gene panel)

Fold change (mean ± SD) relative to DMSO control, normalized to *cdc-42*.

| Category | Gene | Fold change (mean ± SD) | Significance | Adjusted p-value (Šidák) |
|---|---|---|---|---|
| Energy/IIS–FOXO & Longevity Regulators | *aak-2* | 0.99 ± 0.17 | ns | >0.9999 |
| | *daf-2* | 1.14 ± 0.17 | ns | 0.9889 |
| | *daf-16* | 0.99 ± 0.25 | ns | >0.9999 |
| | *skn-1* | 1.09 ± 0.28 | ns | >0.9999 |
| | *sir-2.1* | 1.11 ± 0.15 | ns | 0.9995 |
| Mitochondrial stress adaptation | *atfs-1* | 1.32 ± 0.32 | * | 0.0389 |
| | *hsp-6* | 1.27 ± 0.17 | ns | 0.1716 |
| | *hsp-60* | 1.14 ± 0.08 | ns | 0.9819 |
| | *polg-1* | 1.19 ± 0.22 | ns | 0.7399 |
| | *fmo-2* | 0.91 ± 0.12 | ns | 0.9716 |
| Mitophagy | *pink-1* | 1.12 ± 0.19 | ns | 0.9978 |
| | *pdr-1* | 1.00 ± 0.20 | ns | >0.9999 |
| Stress/Antioxidant defenses | *hsf-1* | 1.07 ± 0.16 | ns | >0.9999 |
| | *hsp-16.2* | 1.24 ± 0.43 | ns | 0.3889 |
| | *gst-4* | 0.95 ± 0.21 | ns | >0.9999 |
| | *ctl-1* | 1.08 ± 0.30 | ns | >0.9999 |
| | *sod-3* | 1.28 ± 0.31 | ns | 0.3445 |
| Lipid metabolism | *acs-2* | 0.95 ± 0.21 | ns | >0.9999 |
| | *nhr-49* | 1.08 ± 0.23 | ns | >0.9999 |
| | *fat-5* | 0.58 ± 0.11 | ** | 0.0012 |
| | *fat-6* | 0.52 ± 0.12 | *** | 0.0001 |
| | *fat-7* | 0.45 ± 0.28 | **** | <0.0001 |

Values represent mean ± SD from five independent biological replicates (n = 5; ~50 µL packed worm pellet per replicate, collected after 48 h treatment). Statistical significance was determined using the 22-gene multiple-comparison framework described in Materials and Methods, with Šidák-adjusted p values. *fat-5*, *fat-6*, *fat-7*, and *atfs-1* were the only genes reaching statistical significance after correction. ns, not significant.

**Supplementary Table S10.** *sod-3*p::GFP and *gst-4*p::GFP quantification statistics

| Reporter | Concentration | Mean fluorescence (% of control) | Significance | Adjusted p-value |
|---|---|---|---|---|
| *sod-3*p::GFP | DMSO 0.5% | 100.00 | — | — |
| *sod-3*p::GFP | 50 µM DHM | 115.0 | ns | 0.3304 |

| Reporter | Concentration | Mean fluorescence (% of control) | Significance | Adjusted p-value |
|---|---|---|---|---|
| *sod-3*p::GFP | 100 µM DHM | 118.0 | ns | 0.2150 |
| *sod-3*p::GFP | 200 µM DHM | 116.7 | ns | 0.2612 |
| *gst-4*p::GFP | DMSO 0.5% | 100.00 | — | — |
| *gst-4*p::GFP | 50 µM DHM | 87.26 | ns | 0.2594 |
| *gst-4*p::GFP | 100 µM DHM | 91.87 | ns | 0.5705 |
| *gst-4*p::GFP | 200 µM DHM | 84.25 | ns | 0.1438 |

Fluorescence intensity was normalized to vehicle (0.5% DMSO) control = 100% for *sod-3*p::GFP and *gst-4*p::GFP. Data represent mean fluorescence from three independent biological replicates (n = 3; at least 30 worms per replicate). Statistical significance was determined by one-way ANOVA with Dunnett's multiple-comparisons test versus vehicle.

**Supplementary Table S11.** SKN-1::GFP subcellular localization (%)

| Concentration | Nuclear (%) | Intermediate (%) | Cytosolic (%) |
|---|---|---|---|
| DMSO 0.5% | 5.72 ± 1.72 | 12.43 ± 2.66 | 81.85 ± 2.95 |
| 50 µM DHM | 2.63 ± 1.78 (ns) | 15.36 ± 1.19 (ns) | 82.01 ± 0.78 (ns) |
| 100 µM DHM | 3.23 ± 0.09 (ns) | 12.34 ± 2.22 (ns) | 84.44 ± 2.16 (ns) |
| 200 µM DHM | 4.59 ± 1.52 (ns) | 15.79 ± 1.95 (ns) | 79.63 ± 2.79 (ns) |

Values are mean ± SD from three independent biological replicates (n = 3). Exploratory two-way ANOVA (concentration × category) detected an interaction ($p = 0.0126$) and localization-category effect ($p < 0.0001$), but no concentration effect ($p > 0.9999$). Dunnett's within-category comparisons detected no difference from vehicle at any DHM concentration (all adjusted $p > 0.05$; 100 µM cytosolic, adjusted $p = 0.2819$). Because the percentages are compositional and sum to 100%, these analyses are exploratory. ns, not significant.

**Supplementary Table S12.** Mitochondrial DNA copy number statistics (related to Section 2.10/3.6)

Relative mtDNA copy number was quantified by qPCR targeting the mitochondrially encoded gene nduo-1 (ND1), normalized to the single-copy nuclear-encoded gene cox-4, in one-day-old adults treated with 100 µM DHM or vehicle (0.5% DMSO) for 48 h. Values represent the fold-change relative to the vehicle-treated mean (set to 1.0) for each of five independent biological replicates.

| Replicate | nduo-1/cox-4 fold-change (normalized to vehicle) |
|---|---|
| 1 | 1.04 |
| 2 | 0.95 |
| 3 | 1.21 |
| 4 | 0.92 |
| 5 | 1.12 |

Mean ± SEM: 1.048 ± 0.054. One-sample t-test against a theoretical value of 1.0: $t(4) = 0.90$, $p = 0.421$ (not significant, n = 5 independent biological replicates).

## Supplementary Figure Legends

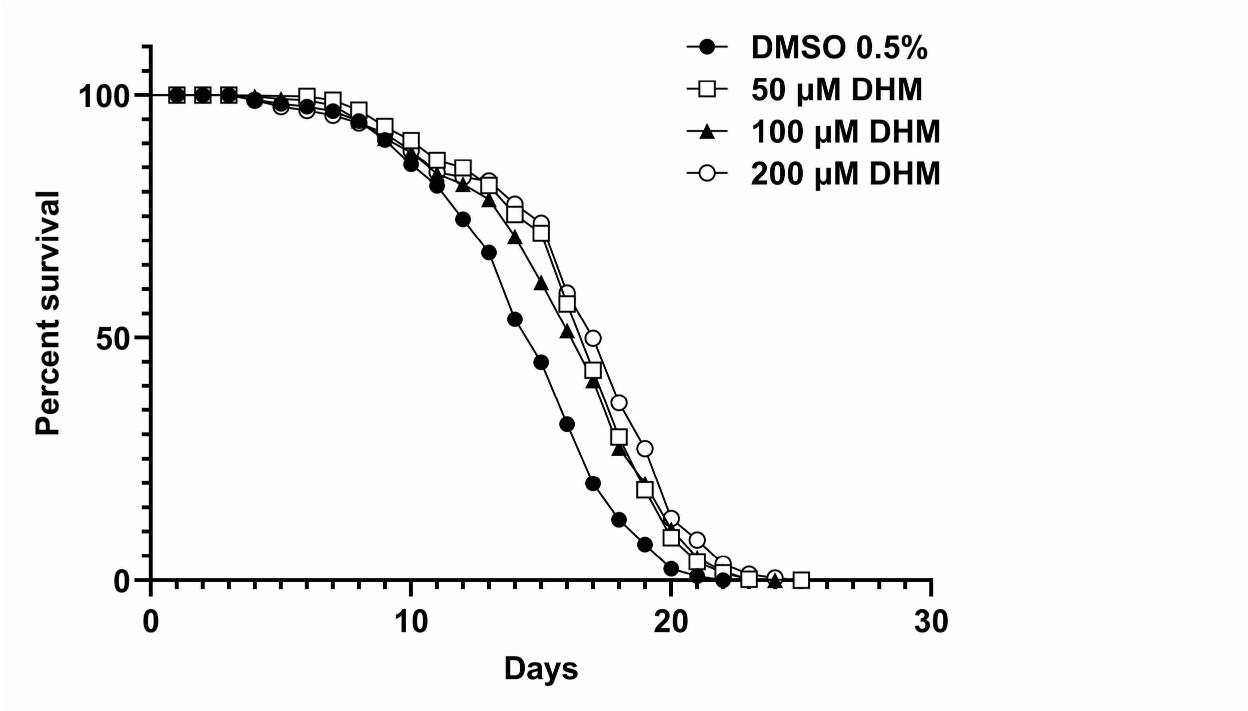


**Figure S1.** Kaplan–Meier survival curves of *daf-16*(mu86) treated with dihydromyricetin. Age-synchronized *daf-16*(mu86) worms were treated with vehicle control (0.5% DMSO) or DHM at 50, 100, or 200 µM. Survival was monitored daily until all worms had died. Curves represent pooled data from three independent assays. Statistical analysis was performed by log-rank (Mantel–Cox) tests. DHM significantly increased survival relative to vehicle at all three concentrations (50 µM: $\chi^2$ = 55.73; 100 µM: $\chi^2$ = 41.64; 200 µM: $\chi^2$ = 84.37; all df = 1, $p < 0.0001$; overall comparison across all four groups: $\chi^2(3)$ = 99.16, $p < 0.0001$). Mean lifespan increased from 14.99 ± 0.22 days (vehicle) to 15.82 ± 0.18, 16.42 ± 0.22, and 16.15 ± 0.21 days at 50, 100, and 200 µM DHM, respectively. N (Day 0): 339 (vehicle), 389 (50 µM DHM), 384 (100 µM DHM), 386 (200 µM DHM); deaths/events: 336, 386, 381, and 383, respectively (3 censored subjects per group).

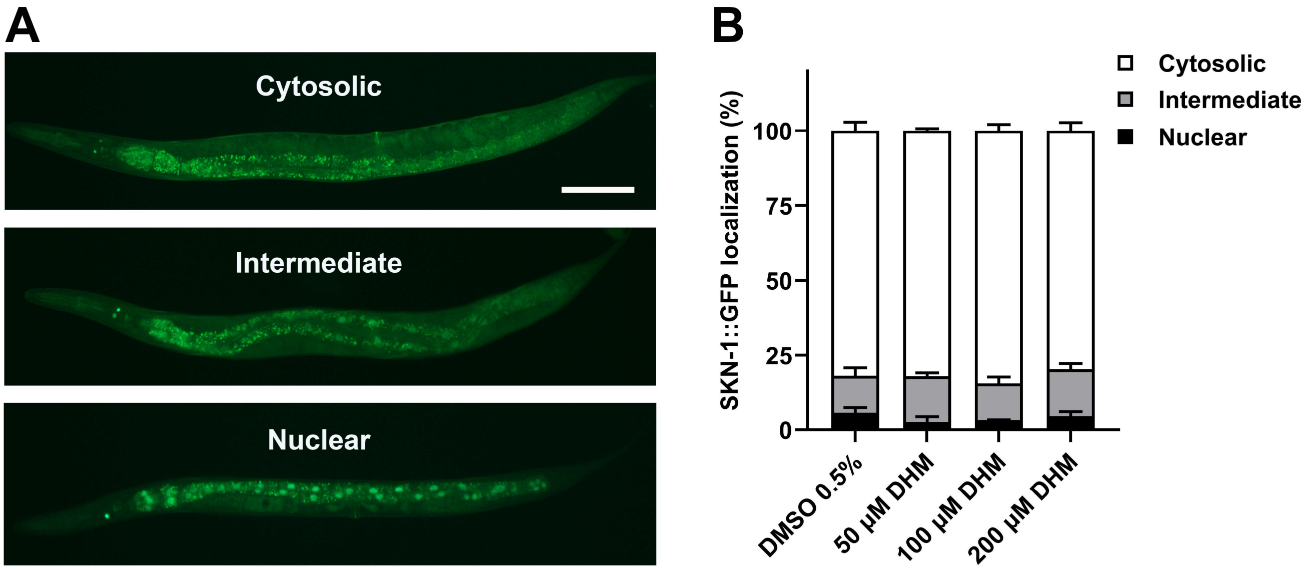


**Figure S2.** SKN-1::GFP subcellular localization following DHM treatment. (A) Representative fluorescence images of LD1 [SKN-1::GFP] worms treated with vehicle or DHM, showing cytosolic, intermediate, and nuclear localization patterns. Scale bar = 100 µm. (B) Quantification after 14 h treatment with vehicle or DHM (50, 100, or 200 µM). Data are mean ± SD from three independent biological replicates. Exploratory two-way ANOVA/Dunnett analysis detected no significant change in any localization category at any DHM concentration (all adjusted $p > 0.05$; Table S11); the percentages are compositional.
